# Combination effect of laser diode for photodynamic therapy with doxycycline on a wistar rat model of periodontitis

**DOI:** 10.1186/s12903-021-01435-0

**Published:** 2021-02-19

**Authors:** Suryani Dyah Astuti, Irawan Budi Utomo, Ernie Maduratna Setiawatie, Miratul Khasanah, Hery Purnobasuki, Deny Arifianto, Kartika Anggraini Alamsyah

**Affiliations:** 1 Department of Physics, Faculty of Science and Technology, Universitas Airlangga, Mulyorejo Street, Campus C Universitas Airlangga, Surabaya, 60115 Indonesia; 2 Biomedical Engineering Master Program, Faculty of Science and Technology, Universitas Airlangga, Surabaya, 60115 Indonesia; 3 Biophysics and Medical Physics Research Group, Faculty of Sciences and Technology, Universitas Airlangga, Surabaya, 60115 Indonesia; 4 Department of Periodontology, Faculty of Dentistry, Universitas Airlangga, Surabaya, 6011 Indonesia; 5 Department of Chemistry, Faculty of Science and Technology, Universitas Airlangga, Surabaya, 60115 Indonesia; 6 Department of Biology, Faculty of Science and Technology, Universitas Airlangga, Surabaya, 60115 Indonesia; 7 Faculty of Voccasional, Universitas Airlangga, Surabaya, 60115 Indonesia

**Keywords:** Diode laser, Photodynamic therapy, Doxycycline, Wistar rat, Periodontitis

## Abstract

**Background:**

Periodontitis is a chronic inflammatory disease characterized by progressive damage on the structure of tooth-supporting tissues. The aim of the study is determining the combination photodynamic effect of diode laser 405 nm treatments and the administration of doxycycline 0.1% within 1, 3, 5, and 7 days on a Wistar rat model of periodontitis.

**Methods:**

Samples were induced with *Porphyromonas gingivalis* ATCC33277 to allow periodontitis development and were treated with combination of doxycycline and laser diode, then statistical analysis was carried out (One-Way ANOVA test and the post-hoc Duncan test; Kruskal–Wallis test and Mann–Whitney follow-up test for non-parametric data). Samples were divided into five groups, laser exposure used was 405-nm diode laser with energy density of 8 J/cm^2^. The expression level of histomorphometric was calculated by measuring the number of macrophages, lymphocytes, fibroblasts and the distance between the CEJ-AV.

**Results:**

The results showed that the combination treatment of doxycycline and laser exposure yielded immunomodulatory effects. The expression level of fibroblast and the distance between CEJ-AV bone showed that the combination of doxycycline and laser therapy exerted healing effect in rat models of periodontitis on day 5 and 7.

**Conclusion:**

The combination of doxycycline 0.1% and diode laser therapy provides a healing effect in rats models of periodontitis.

## Background

Periodontitis is chronic inflammatory disease characterized by progressive damage on the tooth-supporting tissue structure [1]. The damage process depends on the host immune response to the local aggregation of bacteria that causes a variety of biological events that play a role in the achievement of host homeostasis [2]. Damage includes gingival inflammation, erosion of connective tissue attachment, and resorption of the alveolar bone [3]. The resorption in the alveolar bone is mediated by osteoclasts and may cause degradation in the alveolar bone [4]. If such process occurs, it will lead to the disconnection of periodontal ligaments which attached to the alveolar bone, resulting teeth removal from their attachment [5]. Thus, without immediate treatment, this defect will cause more than one tooth loss and even lead to systemic disease.

Periodontitis is known as a disease that is difficult to prevent and cure. Treatment and prevention methods that have been used so far only aim to eliminate bacterial colonies causing infections in the supragingival and subgingival areas [6] so that the tissue can be repaired and regenerated [1, 7]. The mechanical process, either by brushing teeth or the Scale Root Planning (SRP) method, are not effective in removing plaque from the teeth because the probe could not reach a tooth area of > 5 mm [8]. Hence, the use of antibiotics is an effective method for bacterial colonies removal, as stated by the World Health Organization in 2014 [9]. However, long-term application may increase the resistance of bacteria to antibiotics [10]. One alternative treatment modality that would help overcome this problem is photodynamic therapy.

Photodynamic therapy is a non-invasive therapeutic method that utilizes photons of light energy for medical applications [11, 12]. This therapy has been used to treat chronic inflammation and is believed to be an alternative for treating antibiotic-resistant bacteria [9, 13]. The success of the therapy depends on availability of three main components, namely light, photosensitizer, and oxygen [14]. These three components play a vital role in the process of light absorption by photosensitive agents which will activate chemical reactions to produce various reactive oxygen species (ROS) [15, 16]. The various types of ROS produced also play an important role in bacterial photoinactivation since the resulting oxidative effect can inhibit bacterial activity [17] and therefore cause bacterial’s cell death.

Naturally, bacteria produce light sensitive endogenous porphyrin compounds as photosensitizer molecules. Porphyrins produced by bacteria have a very strong absorption in the visible light spectrum, also known as soret peaks located on 405 nm wavelength. The endogenous porphyrin in *Porphyromonas gingivalis* is *dimericiron protoporphyrin IX* (μ-oxobishaem). The choice of photosensitizer for photodynamic therapy in dentistry is highly dependent on the light source used. Laser diode produces light with a range of visible and infrared light spectrum, so it can be applied for periodontitis therapy. Diode lasers are bactericidal and able to accelerate the process of coagulation and regeneration of periodontal tissues [18].

Several studies on photodynamic therapy for the bacteria *Porphyromonas gingivalis* and other periopathogenic bacteria have been reported. The use of blue LED instrumentation and the addition of exogenous photosensitizers with energy density of 16.19 J/cm^2^ caused the inactivation of *S. aureus* bacteria up to 91% [19]. An exposure of *P. gingivalis* with 405 nm diode laser and 4 J/cm^2^ energy density inhibits the growth of *P. gingivalis* bacteria up to 97%, while exposure with energy dose of 8 J/cm^2^ inactivates almost 100% *P. gingivalis* [20]. These researches have proven that the use of visible light diode laser instrumentation, with or without the addition of photosensitizer, can inactivate periopathogenic bacteria in the photodynamic therapy.

The process of bacterial inactivation in photodynamic therapy involving combined use of lasers and antibiotics [21]. The combination of diode laser with antibiotics has been carried out to increase the effectiveness of bacterial inactivation. Doxycycline, an antibiotic type of bacteriostatic tetracycline, plays a role in reducing the formation of polysaccharides [22]. It has been proven to inhibit collagen-destroying enzymes and suppress the growth rate of the bacteria *A. actinomycetemcomitans*. Doxycycline 0.1% also acts as an exogenous photosensitizer for its absorbance in 375–780 nm wavelength. The administration of low-dose antibiotics will minimize adverse effects without causing antibiotic resistance in the bacteria [23]. The results of previous studies showed the ability of low dose doxycycline 0.1% as photosensitization agent combined with a diode laser treatment to reduce bacterial biofilms in vitro [24].

This study aimed to determine the effectiveness of 405-nm diode laser photodynamic therapy with an energy density of 8 J/cm^2^, with and without doxycycline 0.1%, in a Wistar rat model of periodontitis with a variation of four therapies on day 1, 3, 5, and 7. The representative factors that can indicate local changes as the effect of laser are histopathological and histomorphometric of periodontal tissue. Histopathology of wounds is a very helpful tool to exclude a malignancy cause, monitor healing progress in the course of treatment, better understand the pathophysiology of non‐healing wounds, assess morphological changes and help with diagnosis. In this study the expression level of histopathological calculated by measuring the number of macrophages, lymphocytes and fibroblasts. Histomorphometry is broadly defined as the measurement of the shape or form of a tissue. Quantitative analysis of bone architecture is achieved using bone histomorphometry which provides valuable information on the amount of bone and its cellular activity. While the expression level of histomorphometric measurement involved calculating the distance between the CEJ-AV.

## Methods

### Wistar rat model of periodontitis

The experimental animals were purchased from the Faculty of Veterinary Medicine, Airlangga University (acquired from private research source) to ensure that the experimental animals used came from the desired strain. The randomized in vivo treatment including 60 male Wistar rats each that were acclimatized for 7 days and had following characteristics: (1) clinically healthy; (2) age ± 12 weeks; and (3) weight ± 200 g. Ethical considerations used here granted by Animal Care and Use Committee under Faculty of Veterinary Medicine, Airlangga University. Ethical clearance No. 736-KE is in accordance with treatment protocols for experimental animals (see Additional file 5: Ethical clearance). Each rat was induced with *Porphyromonas gingivalis* ATCC33277 sample solution (0.2 ml 1 × 10^9^ CFU/ml) in the gap between the gums and teeth (subgingival area) of the first molars [26]. Bacterial injection was administered using a 1-cc syringe into the subgingival of the left and right lower molar teeth. Experimental animals that were administered the infection were then left for 14 days to allow the development of periodontitis [5].

### Therapeutic administration procedures

At this stage the researchers were careful to prevent several factors that cause discomfort, distress, pain and death in rats.

### Euthanasia procedures for experimental animals and collection of preparations

Sampling and euthanasia of experimental animals was carried out on each group of rats after being given treatment. First, euthanasia was performed on Wistar rats by neck (*cervical*) dislocation. The euthanasia method aims to separate the skull and brain from the spinal cord due to pressure on the skull base. The spinal cord which functions to control respiration and heart activity will be damaged and then the breathing will stop, blocking blood flow which causes death [26]. As soon as the rats have become unresponsive, surgery can be performed. Mandibular tissue can be obtained and incised with a scapel and then put in a 10% neutral formaline buffer solution in the urine pot. After that, the remaining animals were burned in an incinerator.

### Light source

The diode laser (Sony, Japan) emitted a wavelength 405 nm with a stable output power of 30.45 mW, was tested using the Jasco CT-10 monochromator (Jasco, Spain). Spot area focus is 0.152 ± 0.009 cm^2^. Temperature measurements during irradiation showed stability in 32 ± 0.20 °C. The irradiation was performed for 40 s to each target area, delivering 1.22 J of energy with energy density of 8 J/cm^2^. Energy density represents the therapeutic dose administration on target tissue and represent the product of output power and irradiation time, divided by irradiated area. Irradiance is the radiant flux received by some surface per unit area. Laser energy is the output power multiplied by the irradiation time. Energy density was calculated as follows [27]:

$$\begin{aligned} {\text{energy density }}\left( {{\text{J}}/{\text{cm}}^{2} } \right) & = {\text{irradiance}}\,\left( {{\text{W/cm}}^{ - 2} } \right) \times {\text{irradiation time }}\,\left( {\text{s}} \right) \\ {\text{laser energy}}\,\left( {\text{J}} \right) & = {\text{output power}} \left( {\text{W}} \right) \times {\text{irradiation time}} \,\left( {\text{s}} \right) \\ \end{aligned}$$

The laser parameter showed at Table 1 (see Additional file 1: Meta data-1 Characterization of diode laser for more details).

**Table 1:** The parameter of laser diode

Laser parameters	
Parameter	Value
Emitter type	Laser diode
Center wavelength	405 ± 0.07 nm
Operating mode	Continuous wave (CW)
Polarization	Linear
Beam spot size at target	≈ 1.52 ± 0.01 mm^2^
Beam divergence	≈ 12.4° parallel to beam
	≈ 24.8° perpendicular to the beam
Application technique	1 cm perpendicular to tissue
Aperture diameter	0.44 cm
Power	30.45 ± 0.08 mW
Beam shape	Circular
laser exposure time	40 s
Spectral bandwidth	10 nm
energy density	8 J/cm^2^

### Treatment procedure

Samples of 60 wistar rats were divided into the following five groups: Group S, as the non-periodontitis control group without treatment; Group P, the periodontitis group without treatment; Group PL, the periodontitis group with diode laser treatment; Group PD, the periodontitis group with doxycycline treatment; and Group PLD, the periodontitis group with doxycycline treatment combined with diode laser treatment. Each group made of 3 replications and the therapy conducted in 4 time periods, with 2 days interval (day 1, 3, 5 and 7). Diode laser treatment (Group PL) used 405-nm wavelength with energy density 8 J/cm^2^. This therapy was conducted using irradiation beam perpendicular to the periodontitis area within 1 cm distance. Doxycycline treatment (Group PD) was administered using 0.1 g doxycycline diluted in 100 ml distilled water to obtain 0.1% concentration. Next, a microbrush was dipped in the doxycycline solution and applied to the periodontitis area. In the PLD therapy, doxycycline was applied first; then after 30 s, diode laser therapy was performed [28].

### Histopathological analysis of macrophages, lymphocytes and fibroblasts of the periodontal tissue

The expression level of macrophages, lymphocytes, and fibroblasts cells was calculated by measuring the number of cells in each sample. Observations were made using a Nikon Eclipse E-200 (Nikon, Japan) microscope at 400 × magnification.

### Histomorphometric analyses of the distance between the CEJ-AV

Histomorphometric calculation is the calculation approved by the ASBMR; used as one of the parameters to determine the degree of periodontitis pain [4, 5]. Preceding the calculation of CEJ-AV distance, the microscope was calibrated to obtain the units in micrometers (µm). Then, the CEJ-AV distance of the first molar in each sample were measured. Observations were made using a Nikon Eclipse E-200 (Nikon, Japan) microscope at 100 × magnification.

### Statistical analysis

Analysis of the parametric and data distribution normality in this study were performed using One-Way ANOVA and post-hoc Duncan test to obtain the mean difference between each treatment groups. Meanwhile, statistical analysis for non-parametric scale data was performed using the Kruskal–Wallis and Mann–Whitney follow-up test.

## Results

The diode laser used here has peak specification in 379.81 ± 5.11 nm wavelength. The wavelengths are in accordance with the absorption spectrum range of both *Porphyromonas gingivalis* bacteria and doxycycline that were used as exogenous photosensitizers in this study. Previous reports have shown doxycycline absorption spectrum within range 375–380 nm [24]. Lasers with specific power and duration of radiation play an important role in the tissue interaction. In our photodynamic therapy, a photochemical interaction of 30.45 mW low laser power was observed. The temperature generated by diode laser during exposure for 5 min was in the range of 31.20 ± 0.06 °C. The temperature tended to fluctuate during exposure; however, still classified as ambient temperature and therefore did not cause photothermal effects. The radiation density was 8 J/cm^2^, and the exposure time was 110 s.

### The expression level of macrophages, lymphocytes, and fibroblasts cells

The expression levels of macrophages, lymphocytes, and fibroblasts were measured by observing the anatomic histopathology based on the administration of treatment combinations and the amount of therapy. The results of average measurements comparison in each group are presented in Tables 2, 3 and 4 (see Additional file 2: Meta data-2 Histopathology and histomorphometric for more details).

**Table 2:** The expression level of macrophages with the administration of a combination of treatments and various amount of therapy

Group	Day 1		Day 3		Day 5		Day 7	
	Mean	SD	Mean	SD	Mean	SD	Mean	SD
S	5.00	1.00	4.00	1.00	5.00	1.73	5.00	1.00
P	9.33	2.08	7.67	0.58	10.00	2.00	8.67	2.08
PL	6,33	1.53	6.00	0.00	7.00	1.73	5.67	2.08
PD	5.33	0.58	4.67	1.15	5.00	1.73	4.67	2.89
PLD	4.00	0.00	3.00	0.00	3.67	1.15	3.00	1.00

Table 2 shows the expression level of macrophages with the administration of treatment combinations and various level of therapy. Macrophages, lymphocytes and fibroblasts on a nonparametric scale were analyzed by Kruskal–Wallis and the Mann–Whitney follow-up test. Based on the statistical analysis, the expression level of macrophages in Group S has no significant difference on day 1 to day 7, this result was also observed in Group P. However, both these groups were significantly different on each day. Accordingly, a significant difference from day 1 to day 7 was also shown in the Group PLD against Group P. The data obtained showed a decrease in the expression level of macrophage on day 1 to day 7 in Group PLD. Group PL was significantly different from Group P on day 1 and day 3, but not significantly different from Group S on day 1 to day 7. The expression level of macrophages in Group PD was significantly different from that in Group PLD on all days; however, it was only significantly different from Group P on day 3.

Table 3 shows the expression level of lymphocytes with the administration of treatment combinations and various levels of therapy. The statistical analyses of expression level of lymphocytes showed that the expression in Group S were not significantly different, except in comparison between day 3 versus 5 and in day 3 versus 7. In Group P, the level did not differ significantly on each day. However, the lymphocyte expression levels in the normal group and Group P were significantly different from that in Group PLD. High level of expression on each day was shown in the PL and PLD groups with a significant difference between the two on day 5. The highest levels of lymphocyte expression were found on day 3 in Group PL and Group PLD. Meanwhile, Group PLD did not show significant differences in the expression levels on each day. The expression level of lymphocytes in Group PD was highest on day 3 and was significantly different from that in Group P.

**Table 3:** The expression level of lymphocytes with the administration of a combination of treatments and various amount of therapy

Group	Day 1		Day 3		Day 5		Day 7	
	Mean	SD	Mean	SD	Mean	SD	Mean	SD
S	3.67	0.58	3.00	0.00	4.67	0.58	4.00	0.00
P	5.00	1.00	5.33	0.58	5.33	0.58	4.33	0.58
PL	5.33	1.15	8.33	0.58	7.33	0.58	6.67	1.15
PD	7.33	2.52	9.67	2.31	8.33	0.58	8.67	1.53
PLD	9.00	1.73	11.67	3.21	10.33	0.58	10.00	1.73

Table 4 shows the expression level of fibroblasts with the same parameters. In Group S, the fibroblast expression level was not significantly different on day 1 to day 7; however, it was higher than that in the other groups on each day. In contrast, the expression level of fibroblasts in Group P was not significantly different and was lower than that in the other groups on each day. In the treatment groups, the fibroblast expression level in group PLD was not significantly different; however, it was higher than that in the other treatment groups on each day, with the lowest fibroblast expression level on day 1 and the highest on day 5. Despite significant differences occurred with Group P for all days, some in PL and mostly different with PD, Group PLD did not differ significantly with Group S. For Group PD, the fibroblast expression level increased after treatment on day 3 to day 7 with a significance difference when compared to Group P and without a significant difference in comparison to Group S on day 7.

**Table 4:** The expression level of fibroblasts with the administration of a combination of treatments and various amount of therapy

Group	Day 1		Day 3		Day 5		Day 7	
	Mean	SD	Mean	SD	Mean	SD	Mean	SD
S	32.67	4.62	32.67	5.03	34.33	1.53	34.33	3.06
P	18.67	0.58	18.33	2.31	19.33	1.53	21.67	3.21
PL	25.33	1.15	25.67	0.58	28.00	1.00	28.00	2.65
PD	22.00	1.73	21.00	1.73	24.33	0.58	27.67	8.50
PLD	30.00	4.00	30.67	0.58	34.00	3.61	32.33	3.06

**Table 5:** Comparison of the Cemento Enamel Junction and the alveolar bone (CEJ-AV) distance in each treatment group

Group	Day 1		Day 3		Day 5		Day 7	
	Mean	SD	Mean	SD	Mean	SD	Mean	SD
S	513.01^a,b^	156.11	497.88^a^	101.30	508.91^a,b^	41.06	461.56^a^	41.06
P	893.61^a,b,c,d^	316.78	1.123.23^d^	170.60	964.54^b,c,d^	208.00	995.54^c,d^	34.78
PL	569.25^a,b,c^	87.74	780.78^a,b,c,d^	255.71	662.65^a,b,c^	58.83	644.97^a,b,c^	52.50
PD	759.38^a,b,c,d^	82.08	582.26^a,b,c^	44.28	680.14^a,b,c,d^	185.03	797.25^a,b,c,d^	91.49
PLD	695.96^a,b,c,d^	53.56	695.95^a,b,c,d^	52.66	603.14^a,b,c^	51.65	538.27^a,b,c^	49.53

Note: Index after Mean on the data represent by the same superscript shows no significant difference from the Duncan test results

### The expression level of the Cemento Enamel Junction (CEJ) and the alveolar bone (AV) of the periodontal tissue

The first analysis is the distance calculation between the Cemento Enamel Junction (CEJ) and the alveolar bone (AV). This analysis is also called the histomorphometric analysis and has been approved by the ASBMR committee [29]. Table 5 shows the comparison of the CEJ-AV distance in each treatment group. The CEJ-AV data from the research results were analyzed using the one way ANOVA test with the Statistical Package for the Social Sciences or Statistical Product and Service Solutions (SPSS) version 21 program. The test performed including data normality and homogeneity (with Kolomogorov-Smirnov and Levene's Test). The test results showed data were normally distributed and homogeneous with *p* = 0.547 and *p* = 0.67 > α = 0.05 respectively. Furthermore, the One-Way ANOVA test was carried out to determine the differences in each treatment. The analysis showed that there were significant differences between treatments (*p* = 0 < α = 0.05). Then the post hoc test was carried out using the Duncan test to see the average difference between groups. The statistical analysis of CEJ-AV bone distance in S group showed there were a slight difference, while in P group showed a slight increase but were not significantly different (see Additional file 3: Meta data-3 Statistical analysis for more details).

**Fig. 1:**
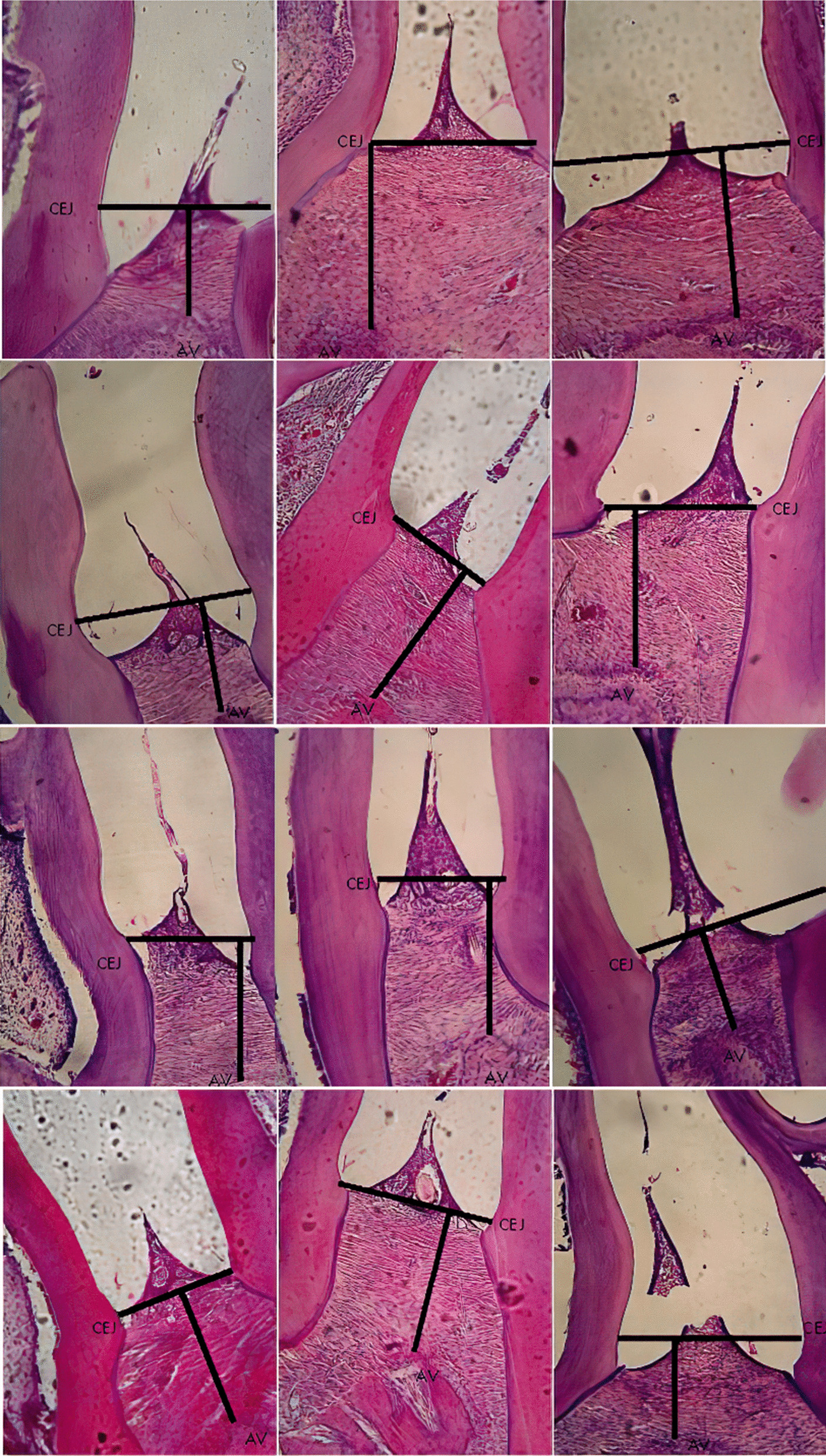
Histomorphometric images of the distance between Cemento Enamel Junction (CEJ) and alveolar bone (AV) on Group Healthy (S) from day-1; 3; 5; and 7 with Haematoxylin and Eosin (H&E) Staining, × 400 magnification

**Fig. 2:**
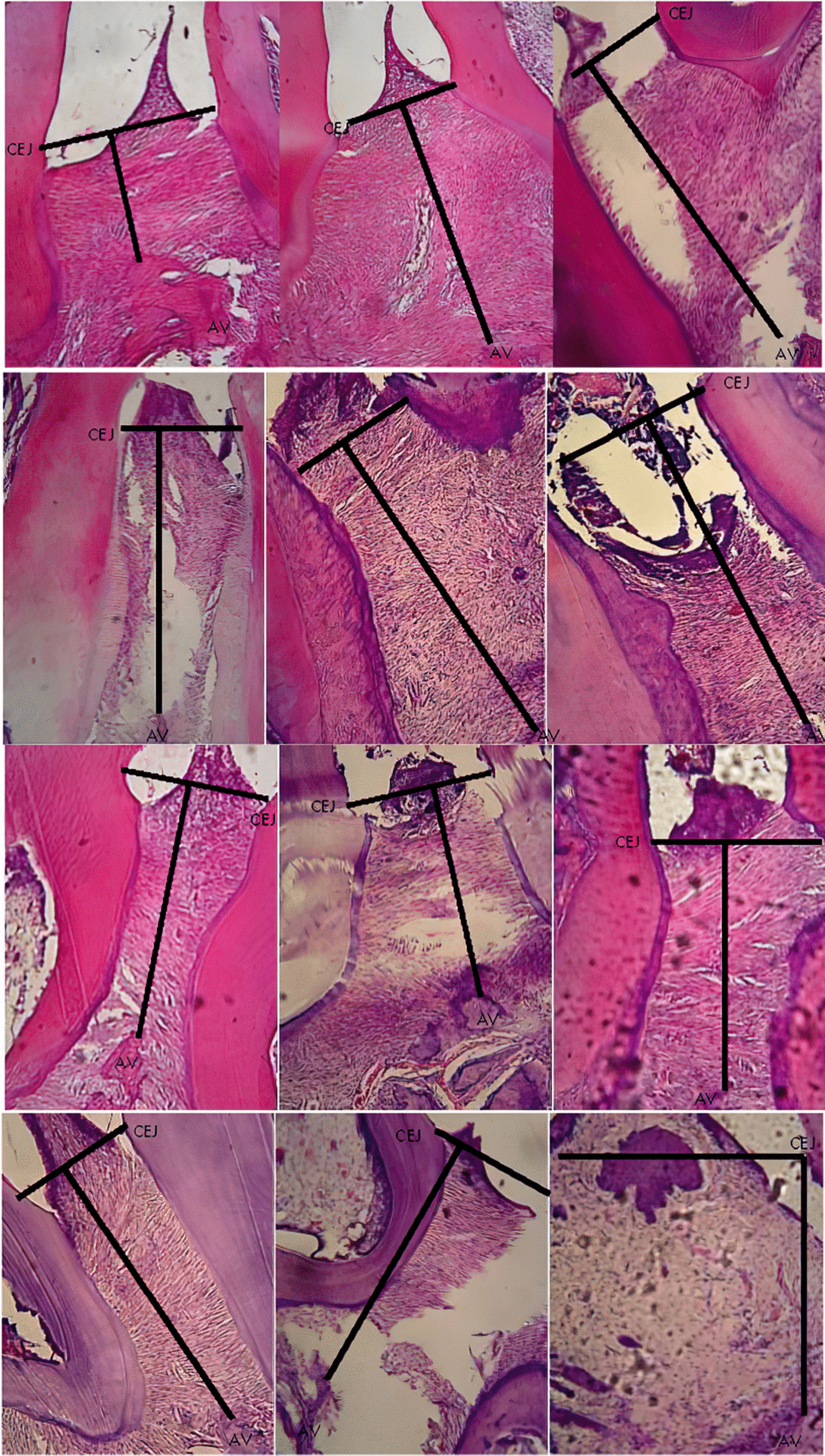
Histomorphometric images of the distance between Cemento Enamel Junction (CEJ) and alveolar bone (AV) on Group Periodontitis (P) from day-1; 3; 5; and 7 with Haematoxylin and Eosin (H&E) Staining, × 400 magnification

**Fig. 3:**
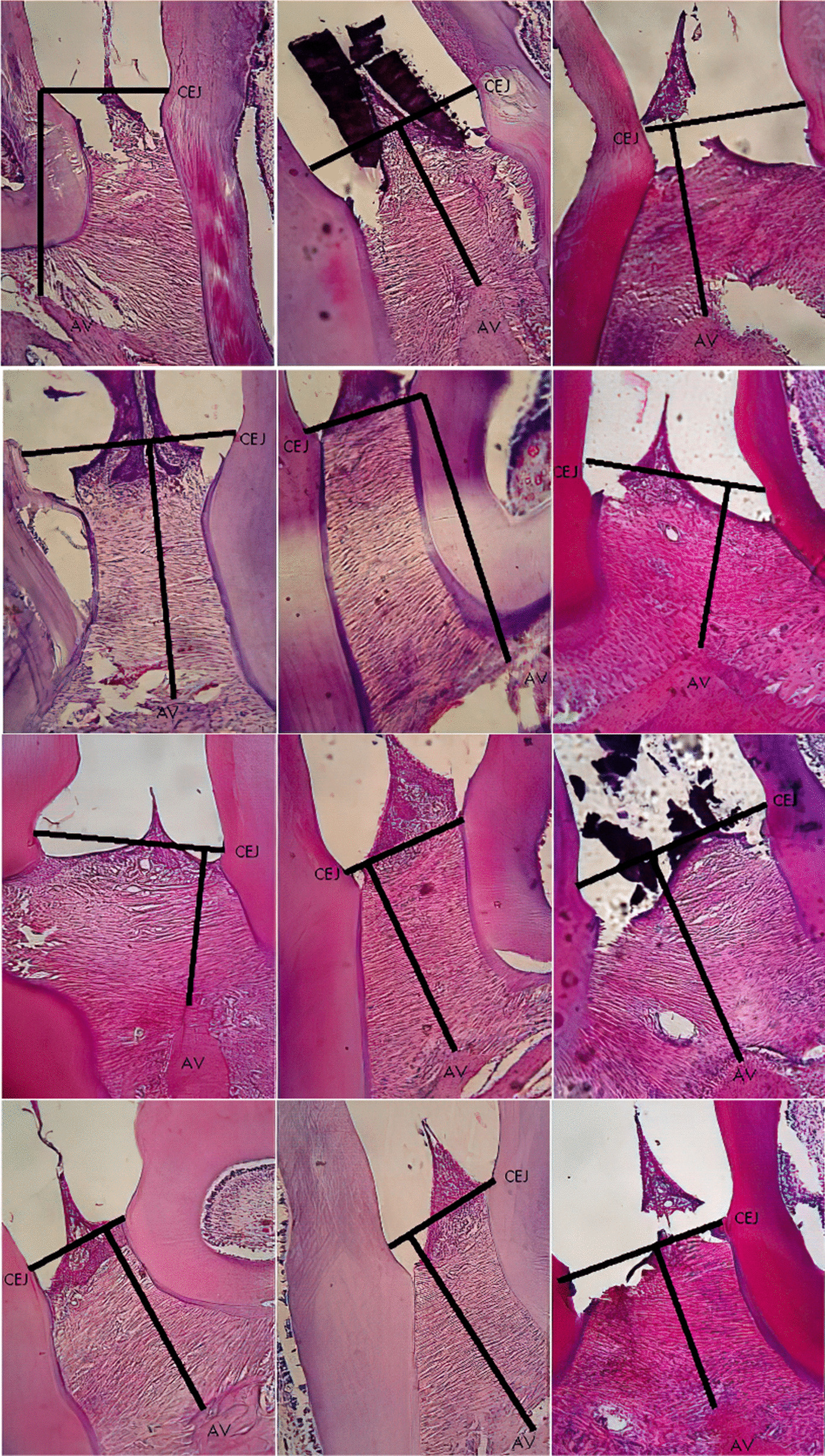
Histomorphometric images of the distance between Cemento Enamel Junction (CEJ) and alveolar bone (AV) on Group Diode Laser treatment (PL) from day-1; 3; 5; and 7 with Haematoxylin and Eosin (H&E) Staining, × 400 magnification

**Fig. 4:**
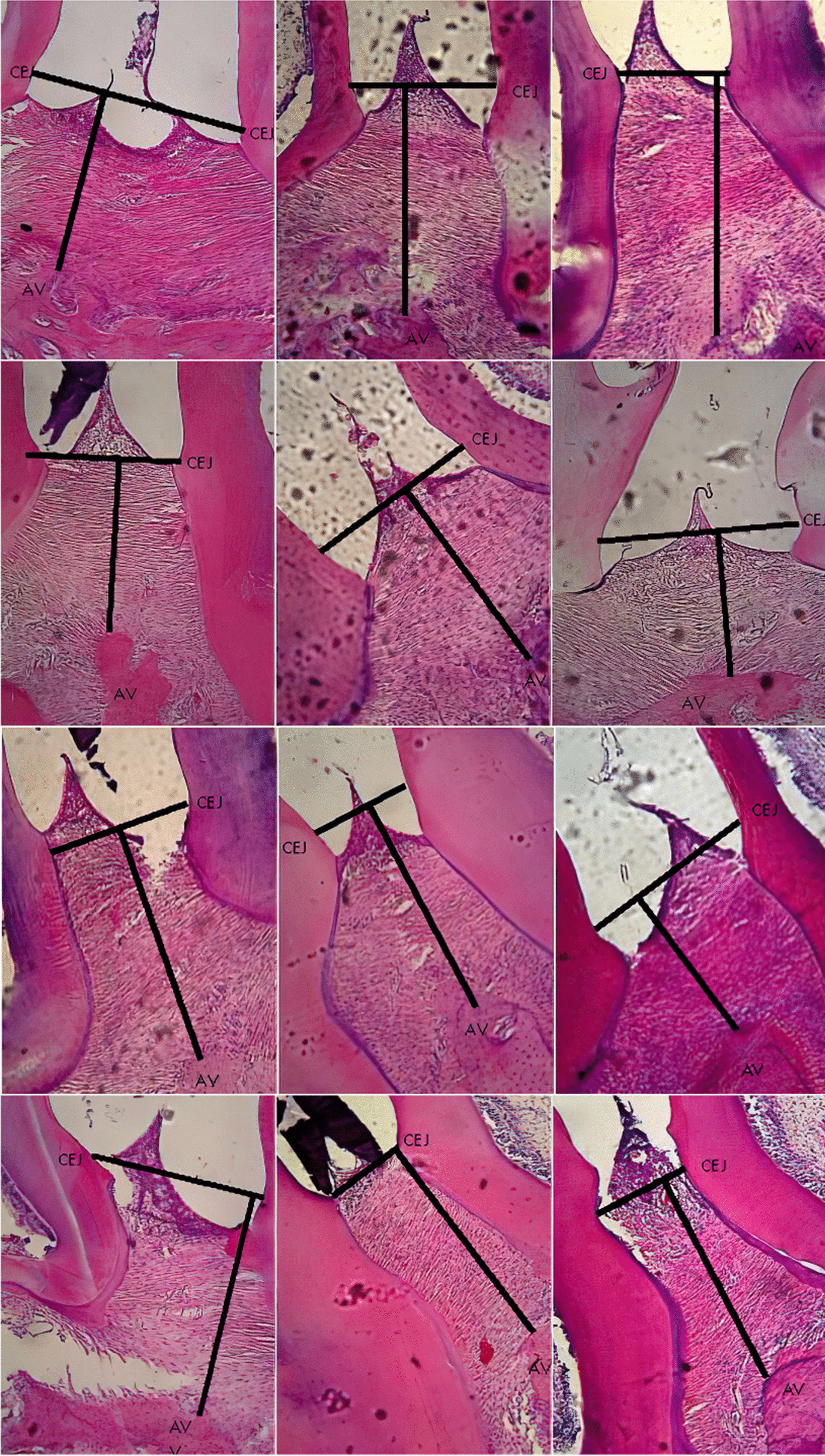
Histomorphometric images of the distance between Cemento Enamel Junction (CEJ) and alveolar bone (AV) on Group Doxycyclin treatment (PD) from day-1; 3; 5; and 7 with Haematoxylin and Eosin (H&E) Staining, × 400 magnification

**Fig. 5:**
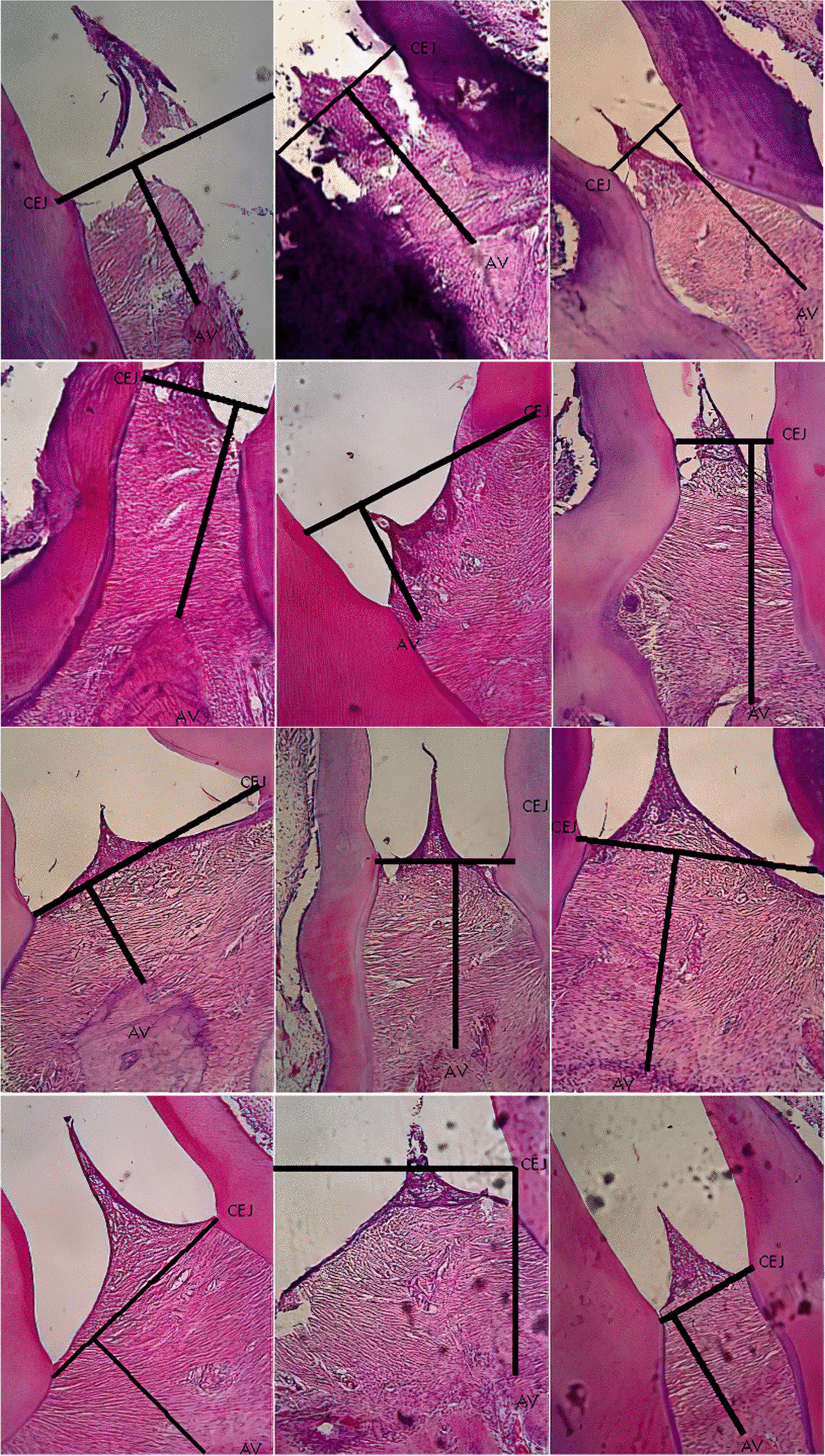
Histomorphometric images of the distance between Cemento Enamel Junction (CEJ) and alveolar bone (AV) on Group Diode Laser-Doxycyclin treatment (PLD) from day-1; 3; 5; and 7 with Haematoxylin and Eosin (H&E) Staining, × 400 magnification

Significant difference observed in the CEJ-AV distance for PL group on day 1, 5 and 7, PD group on day 3 and PLD group on day 5 and 7 compared to control, S group. Histomorphometric analysis images showing an association of the CEJ-AV distance with an increased degree of periodontitis pain [4, 5]. Figures 1, 2, 3, 4 and 5 shows histomorphometric images of the distance between CEJ-AV on day 1, 3, 5 and 7.

Table 4 shows that were a slight increase in the degree of periodontitis in the doxycycline group but no significant difference. Doxycycline is a type of tetracycline that works by inhibiting bacterial activity through the 30 s ribosome, called bacteriostatic; however, it does not inactivate the bacteria. *Porphyromonas gingivalis* has been reported as a bacterium that causes damage to the bone tissue through LPS virulence factors that inhibit osteoblast differentiation [8]. Alveolar bone damage in the periodontal tissue is characterized by bone resorption in the bone tissue, known as resorption bays (lacuna howship) [30]. Osteoclasts, cells that cause bone resorption, are branched motile cells with many nuclei; they are usually located in the lacuna howship [31]. Greater damage to alveolar bone can be interpreted as an increase in the degree of pain in periodontitis that may lead to tooth decay. The administration of doxycycline and diode laser treatment lower the degree of pain in each treatment on day 1 to day 7, as indicated by the decreasing CEJ-AV distance observed here.

**Fig. 6:**
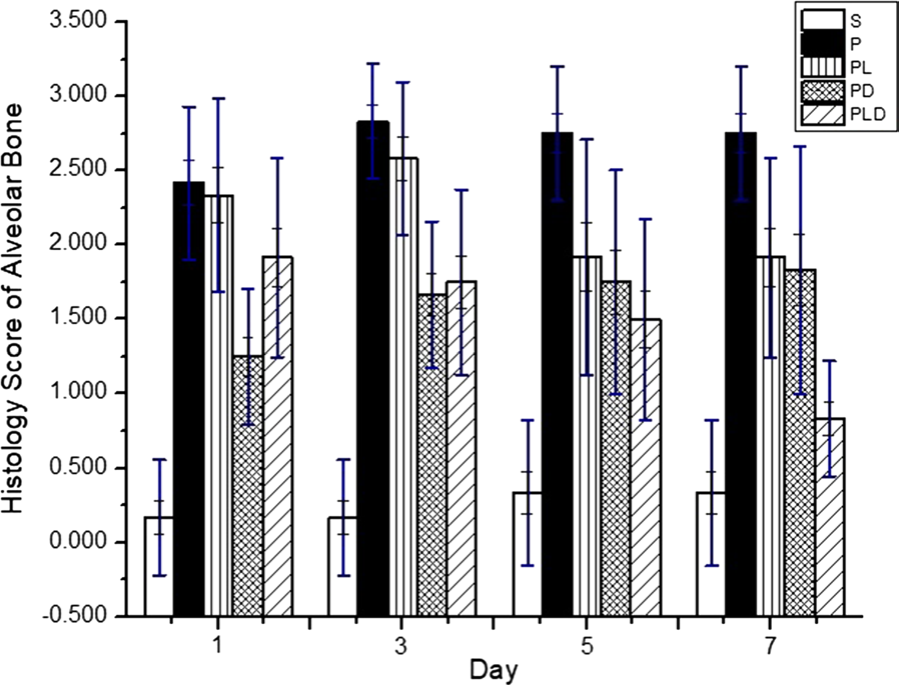
Comparison chart of the histology scores of the alveolar bones in each treatment group

Figure 6 shows the histopathological image of the alveolar bone in rats. This figure show there were no morphological changes in the periodontal tissue of the control (S/healthy) rat group. As predicted, the periodontitis rats group showed damage in the periodontal tissue structure, both in the periodontal ligament and the alveolar bone. Moreover, in this group, we observed damage in periodontal pockets of the alveolar bone, and loss of most of the alveolar bone structure, characterized by chronic periodontitis on day 7 [4, 5].

Figure 6 also depicts the histology scores of alveolar bones in each treatment group. A large amount of lacuna howship was observed in the groups of periodontis rats, including the negative control group, and those treated with diode laser only as well as doxycycline only. It is worth noting that the doxycycline treatment group had an increased alveolar bone resorption score, consistent with the increasing of CEJ-AV distance (Table 4). The alveolar bone resorption score increase indicates alveolar bone degradation and decrease of alveolar bone density, thus lead to CEJ-AV increasing distance [32]. Contrarily, the doxycycline treatment group combined with a diode laser indicates alveolar bone tissue repair (bone remodeling), proven by the CEJ-AV distance decrease (see Additional file 4: Meta data-4 Raw Data of Histology Score for more details).

## Discussion

Laser diode produces light spectrum within range of visible and infrared light, making it applicable for periodontitis therapy. Laser treatment given to periodontitis rats have shown antibacterial effects, indicated by the level of macrophage expression which is not significantly different compared to healthy rats. The lymphocyte expression level tended to be higher than the periodontitis group with significant differences observed on the third, fifth, and seventh day. Fibroblasts component increased after the initial administration of therapy, therefore did not differ significantly in the healthy group on the seventh day. Alveolar bone tissue repair observed after administration by fifth day, marked with CEJ-AV distance decrease during fifth and seventh days. First day administration of laser therapy showed that the CEJ-AV distance was not different from the healthy group. Various research results show that the laser acts as a stimulator that triggers the *de-epitheliazation* process and increases vascular permeability [12]. Both processes are known to inhibit growth factor down-regulation and increase the infiltration of immunocompetent cells [33]. In addition, lasers have been known to trigger the release of various pro and anti-inflammatory mediators such as IL-1, IL-2, IL-6, IL-8, IL-10, IL-1β, TNFα, and G-CSF [32, 34, 35].

There was a significant difference between the healthy rat group and the periodontitis rat group based on calculation in expression level of histopathological analysis. The expression level of macrophages and lymphocytes in the healthy rat group was lower compared to other group. However, the fibroblasts expression level was significantly higher than periodontitis group. Lipopolysaccharides along with other virulence factors are known to have the ability to modulate the host defense system that affects immune down-regulation [25]. The failure of innate-immunity in antigens elimination affects the adaptive-immunity system sustainability [34]. In this condition, macrophages also act as the antigen-presenting cell components, capable of recognizing bacteria and releasing inflammatory mediators [8]. B lymphocytes are involved in bacterial resistance through antigen–antibody reaction, while T lymphocytes mediate host defenses against responses in forming T helper (CD4 + Th) and T cytotoxic (CD8 + cytotoxic) subsets [31]. Failure to overcome the presence of antigens causes the release of various pro-inflammatory mediators, such as IL-1β and TNFα, furthermore, leading to tissue damage [36]. The high expression of macrophages and other proinflammatory mediators can be associated with tissue damage occurrence [5].

Histomorphometric observation results showed a significant increase in the CEJ-AV distance in the healthy group and the periodontitis group. This increase is believed to be the result of bone resorption indicator that occurs in the alveolar bone and become popular method used by many researchers [4, 5, 30].

Bone resorption is one of the physiological responses to prevent bacterial infection using osteoclasts [31]. The differentiation, activation, and survival ability of an osteoclast is influenced by RANKL and osteoprotegrin produced by M_2_ macrophage components [32]. Osteoprotegrin is also produced from the release of IL-10 cytokines by Th_2_ lymphocytes [34]. If RANKL binds more M1 macrophage RANK receptor, then M1 macrophages produces inflammatory mediators to activate the osteoclasts into maturity [30]. On the other hand, if osteoprotegrin can bind more RANKL, fibroblast components can be activated through the production of various mediators growth factor (GF) [35]. This mediator will activate osteoblasts that influence bone tissue remodeling as well as fibroblasts in the formation of collagen matrix. Alveolar bone tissue that has been repaired is known as woven bond (small bones) and can be associated with a decrease in the CEJ-AV distance [31].

The use of diode lasers in the photodynamic therapy is recognized as a therapeutic modality in the bacterial inactivation process. Various reactive oxygen species are produced in the photophysical process through suitability of hematogenphyrin porphyrin absorption spectrum in *P. gingivalis* bacteria [36]. In addition to providing an antibacterial effect, this research demonstrates the role of laser as a therapy that affects the host immune system in cases of chronic inflammation, referred to as immunomodulatory therapy. Photodynamic therapy is believed to have no effect on human gingival fibroblast tissue (HGF) through the elimination of photobiological effects [37]. Furthermore, lasers act as the stimulators that trigger de-epitheliazation and increase vascular permeability [38]. Both these processes inhibit growth factor down-regulation and increase the infiltration of immunocompetent cells.

Doxycycline antibiotics given to the periodontitis rats have shown antibacterial effect. This is demonstrated by an increase in the lymphocyte expression and inversely, a decrease in the macrophage expression on day 3, significantly different from periodontitis rat group. Increased fibroblast expression was observed following the administration of doxycycline on day 5 that continued to rise on day 7 with no significant difference in comparison to the healthy rat group. According to the histomorphometric analysis, the CEJ-AV distance following the administration of therapy on day 3 was not different from healthy rats. However, the distance was distinctive compared to periodontitis rats. Meanwhile, we observed increasing CEJ-AV distance until day 7. Moreover, the expression level of each immunocompetent cell indicates tissue repair, especially shown in macrophages cells decrease as well as increasing of fibroblasts number.

Photodynamic therapy with the addition of exogenous photosensitizer elements has been widely used and increased the effectiveness of therapy, especially in the inactivation of bacteria [24, 27]. In addition, the use of low-dose antibiotics combined with photodynamic therapy aim to avoid increasing bacterial resistance to antibiotics [19]. The suitability of doxycycline absorption spectrum causes photosensitizer molecules to be excited, thus generating the type 1 and type 2 photochemical effects. Various free radicals, especially peroxide compounds, have an impact on bacterial death. The diode laser indirectly acts as an immune system modulator. If the presence of antigens can be overcome, macrophage components will undergo lysis so that their quantity depleted. Thus, it can be assumed that the reduction in proinflammatory mediators and the increase in anti-inflammatory mediators induce tissue repair. Increased expression level of fibroblasts that play a role in the new extracellular matrix also supports tissue re-modelling [30].

The periodontitis manifestations depend on response between the host defense tissue with pathogenic bacteria which involves innate and adaptive immunity [34]. Innate immunity is the host's initial defense system against bacteria that works quickly, but has low specificity and diversity. If the bacteria (antigen) activity cannot be resolved, the adaptive immunity response will automatically begin to work. On the other side, the adaptive immunity response is a specific immune response to an antigen, divided into two types of responses, namely cell-mediated immunity (cellular immunity) and humoral immunity. Cellular immunity is a response that works with specific antigens and induces apoptosis, while humoral immunity is a response which regulates antibodies against foreign antigens. The two immune system responses work collaboratively returning the inflamed tissue to homeostasis [39].

The response mechanism to pathogenic bacteria in periodontal tissue begins with the response of several leukocyte and endothelial cell infiltrations [34]. In this phase, the metabolic products of bacteria, such as lipopolysaccharides, induce the tissue to produce cytokines and neuropeptides which cause blood vessels vasodilation. The released neutrophils leave the blood vessels and target the inflamed area in response to chemokines. In the early lesion phase, the number of neutrophils increases in connective tissue and marked by presence of several leukocyte cells such as macrophages, lymphocytes, plasma cells, and mast cells. The transition phase from innate immunity to humoral immunity occurs in the established lesion phase which is dominated by macrophages, plasma cells, and B and T lymphocytes. In addition, in this phase fibroblasts begin to produce collagen which repairs blood vessels. The transition from gingivitis to periodontitis is the last phase (advanced lesion) which is characterized by periodontal pocket erosion that deepens to alveolar bone destruction and observe-able histologically and clinically. In this phase there is an extension of the infiltration process of inflammatory cells which is also characterized by reduced collagen; as well as increase in number of lymphocytes, plasma cells, and macrophages that play an active role in chronic inflammation. Inflammation is the first physiological response mechanism as a host defense system against local bacterial aggregation in tissue [34]. Inflammation can be described by means of inflammation, resolution, and healing that involve several components of the immune system that interact to protect periodontal tissue. Some of these important components can be explained by their roles and functions as follows:

- Lymphocytes are one type of leukocyte cells that play an important role in the body's defense system against antigens, especially and consist of B cells, T cells, and Natural Killer cells (NK-cells). B cells differentiated in the bone marrow and work by producing antibodies, whereas, T cells are differentiated in the thymus gland and act against specific antigens by producing T helper and also T cytotoxic. At the cellular level, if the number of lymphocytes decreases, the body's ability to deal with antigens, such as bacteria, will decrease [40].
- Macrophages are a type of white blood cell and are the adult forms of monocytes that can migrate through blood vessels. Macrophages (macros and phagens) play an important role in the innate immunity system, which works by recognizing foreign antigens with toll-like receptors (TLRs) and consuming foreign antigens (phagocytosis). Macrophages are said to be the main agents that produce metabolic secretions that cause damage to connective tissue to alveolar bone damage [41]. Macrophages play a role in the regulation of inflammation that occurs, depending on their function, macrophages can produce pro or anti-inflammatory mediators.
- Fibroblasts are the most common cells found in connective tissue and play a role in the tissue repair process through the synthesis of extracellular matrix components, one of which is the process of rebuilding blood vessels (angiogenesis) [30]. Fibroblasts will be active when the host defense system is damaged and so they will proliferate (fibrogenesis). The processes of fibrogenesis and angiogenesis are regulators for determining the function of macrophages.

In this study, researchers had limited information about the antibacterial effects produced through photodynamic therapy using diode lasers combined with doxycycline photosensitizers. Several studies in the use of photodynamic therapy that have been performed on rat with periodontitis have used more specific measurement methods, such as radiological features for measuring the CEJ-AV distance. In addition, specific pro/anti-inflammatory cytokines were used for determining the extent of tissue damage such as IL-1β, IL-6, PGE_2_, TNFα and repair of VEGF, HGF, COL-1A tissues have been conducted, although a different therapeutic method was used.

## Conclusion

The results showed that the combination treatment of doxycycline 0.1% and laser exposure yielded immunomodulatory effects. The expression level of macrophage cells decreased from day 1 (5.00 ± 1.00) to day 7 (2.00 ± 0.00) and the highest level of lymphocyte cells was on day 3 (11.67 ± 3.21). The healing process that is mediated by the expression level of fibroblast is similar to that in healthy rats, especially on day 5 of the therapy (34.00 ± 3.61). The histomorphological analysis performed by measuring the distance between CEJ-AV bone resulting 538.27 ± 88.94 µm, showed that the combination of doxycycline and laser therapy exerted healing effect in rat models of periodontitis on day 7. Based on immunocompetent cells and the histomorphological analysis that measured the macrophage cells, lymphocyte cells, fibroblast and CEJ-AV distance, it can be concluded that the combination of doxycycline and laser therapy provide a healing effect in rats models of periodontitis.

## Supplementary Information

**Additional file 1.** Meta data-1 Characterization of diode laser.

**Additional file 2.** Meta data-2 Histopathology and histomorphometric.

**Additional file 3.** Meta data-3 Statistical analysis.

**Additional file 4.** Meta data-4 Raw Data of Histology Score.

**Additional file 5.** Ethical clearance.

## Data Availability

The datasets analyzed during the current study are available from the corresponding author on reasonable request.

## Abbreviations

CEJ-AV: Cemento Enamel Junction and the alveolar
ROS: Reactive oxygen species
