# Supplementary material for: Combination effect of laser diode for photodynamic therapy with doxycycline on a wistar rat model of periodontitis
Source: BMC Oral Health. 2021 Feb 19;21:80. doi: 10.1186/s12903-021-01435-0 (PMC7893773; doi:10.1186/s12903-021-01435-0)

Diode Laser Characterization

The beam profile of the diode laser:


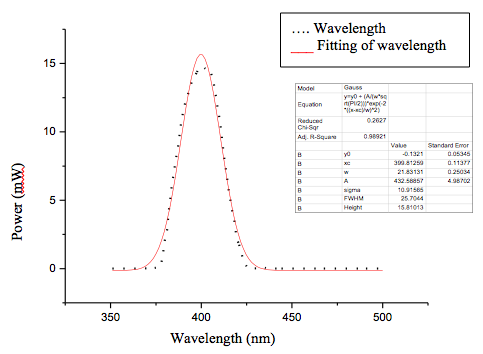


The beam divergence of the diode laser is:


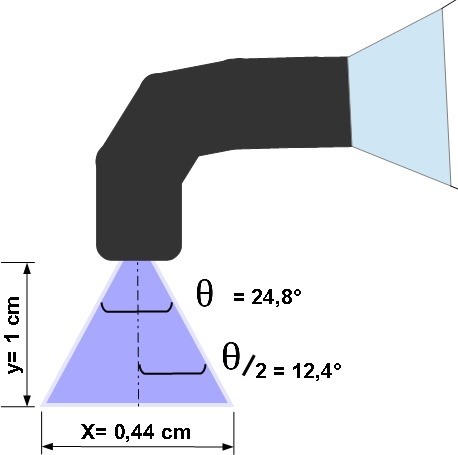


Stability of diode laser


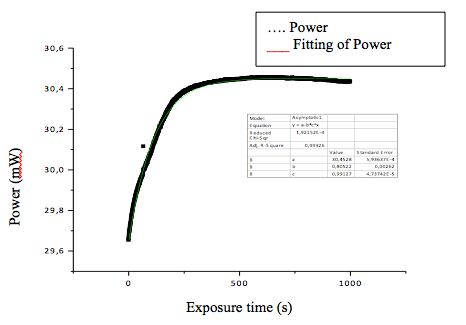


The temperature of exposure time:


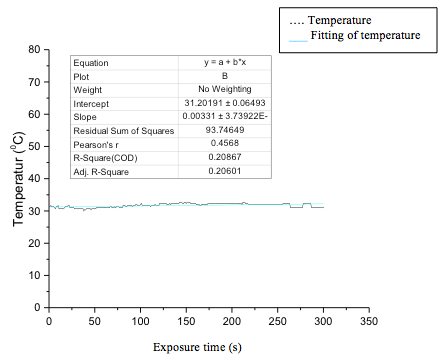


The parameter of Laser Diode

| **Laser parameters** |  |
| --- | --- |
| Parameter | Value |
| Emitter type | Laser Diode |
| Center wavelength | 405 ± 0,07 nm |
| Operating mode | Continuous wave (CW) |
| Polarization | Linear |
| Beam spot size at target | ≈ 1.52 ± 0.01 mm^2^ |
| Beam divergence | ≈ 12.4^o^ parallel to beam  ≈ 24.8^o^ perpendicular to the beam |
| Application technique | 1 cm perpendicular to tissue |
| Aperture diameter | 0.44 cm |
| Power | 30.45 ± 0,08 mW |
| Beam shape | circular |
| laser exposure time | 40 s |
| Spectral bandwidth | 10 nm |
| energy density | 8 J/cm^2^ |

The absorption spectrum of doxycycline


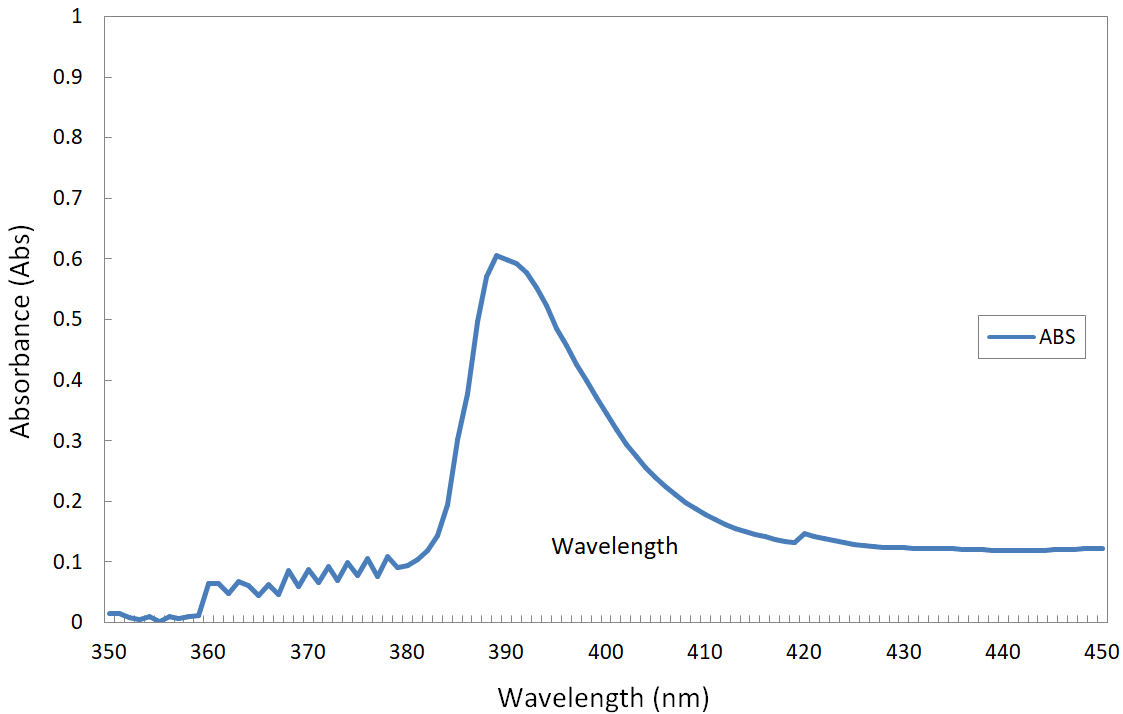

Supplement: Supplementary file 1 — Additional file 1. Meta data-1 Characterization of diode laser. [file 12903_2021_1435_MOESM1_ESM.docx]
