# Supplementary material for: Combination effect of laser diode for photodynamic therapy with doxycycline on a wistar rat model of periodontitis
Source: BMC Oral Health. 2021 Feb 19;21:80. doi: 10.1186/s12903-021-01435-0 (PMC7893773; doi:10.1186/s12903-021-01435-0)
Supplement: Supplementary file 2 — Additional file 2. Meta data-2 Histopathology and histomorphometric. [file 12903_2021_1435_MOESM2_ESM.doc]

**Histopathology of Alveolar Bone**

**NORMAL**


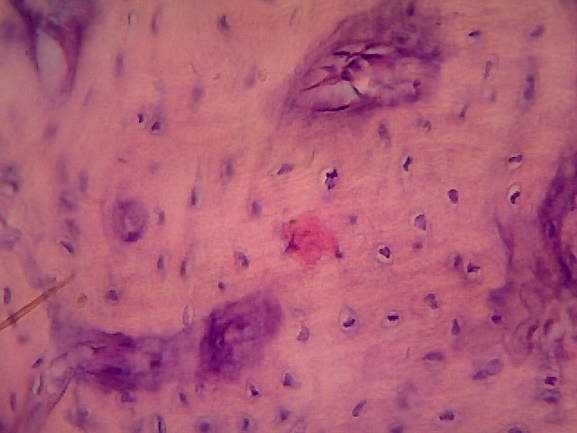

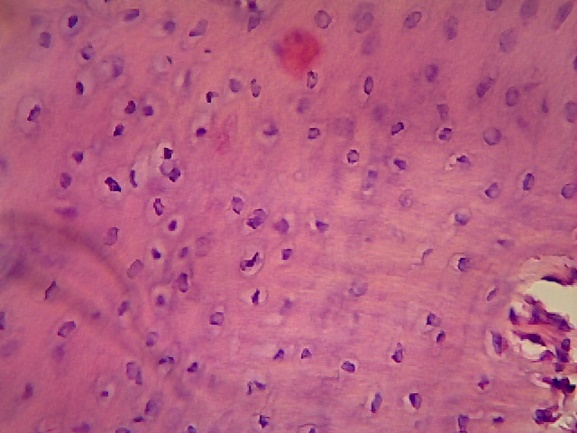


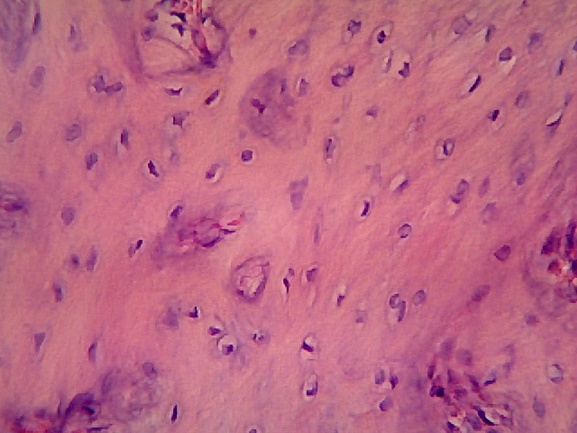

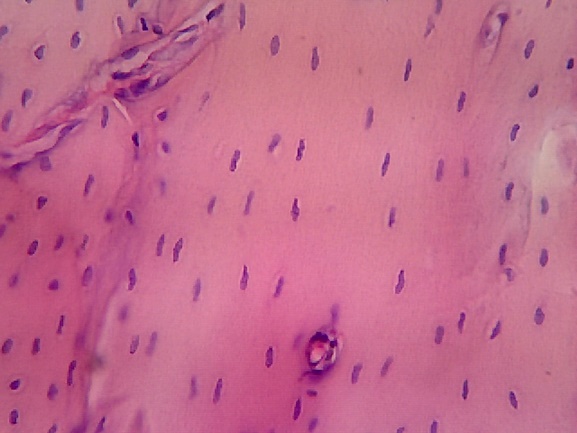


A1a


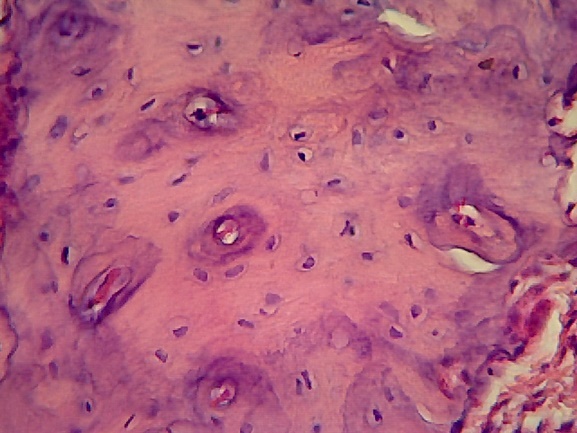

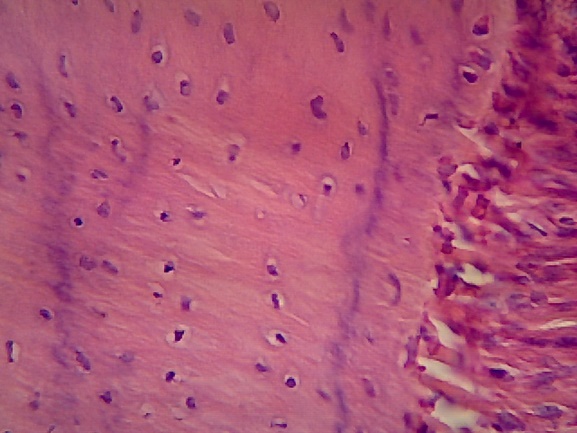


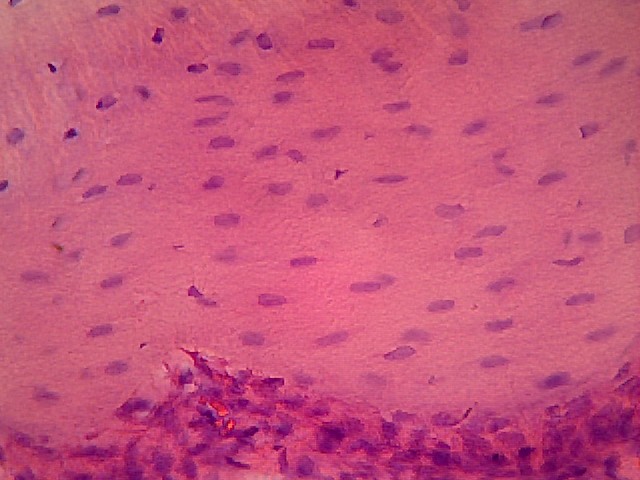

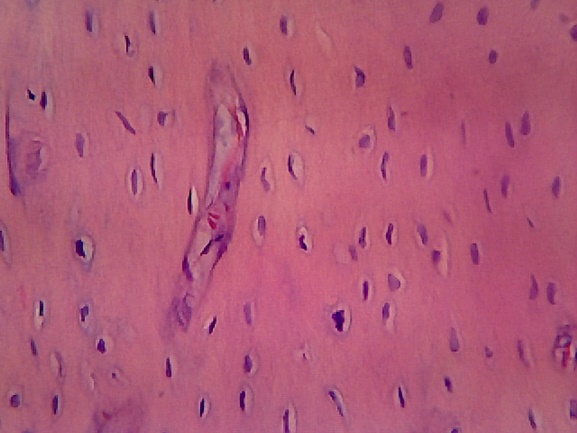


A1b

**
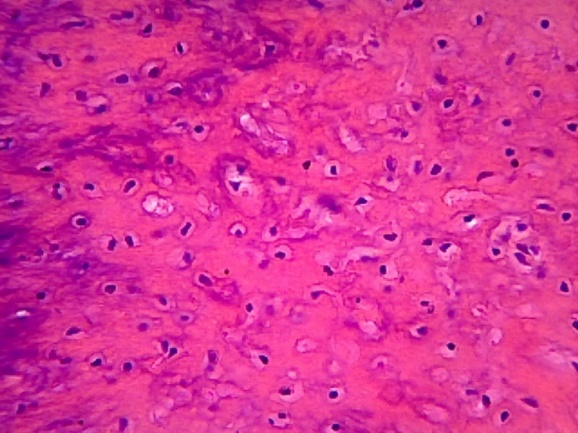
**
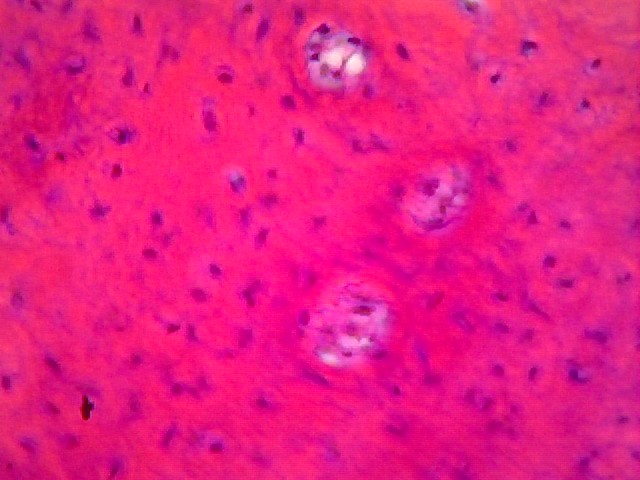


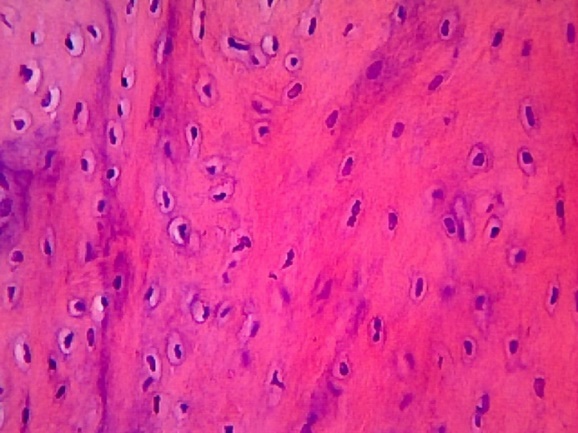

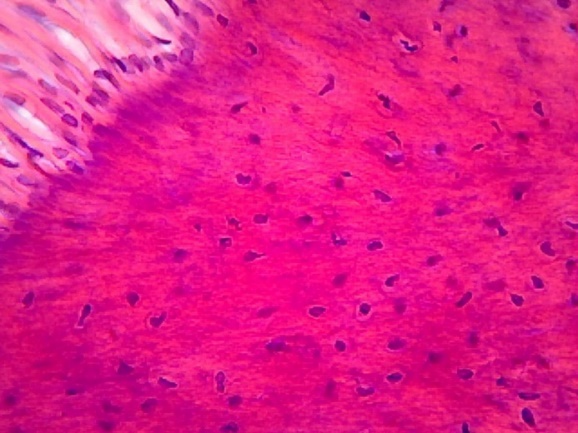


A1c


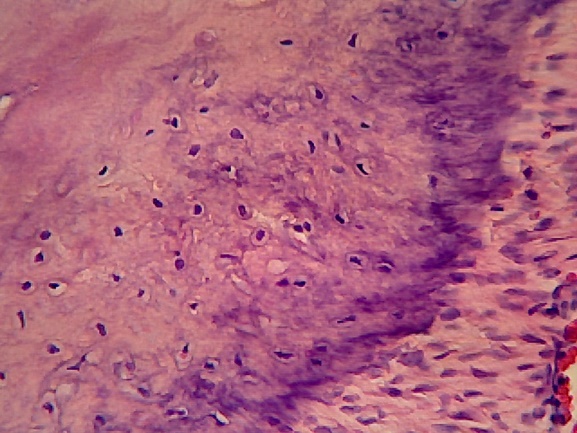

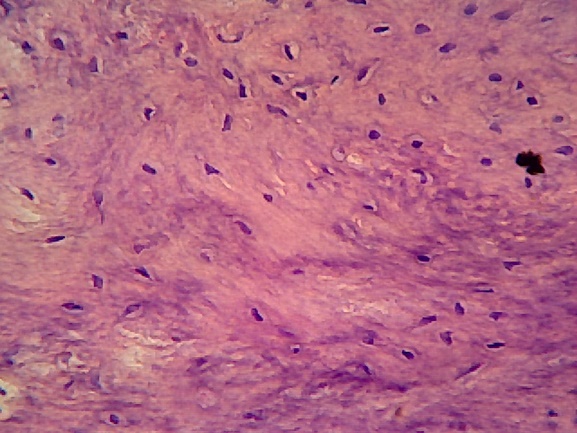


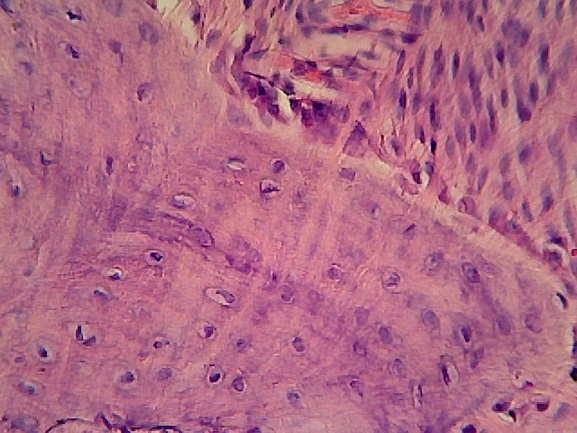

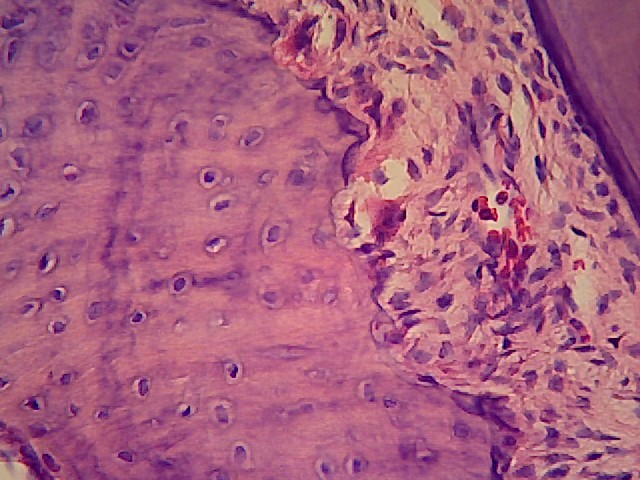


A2a


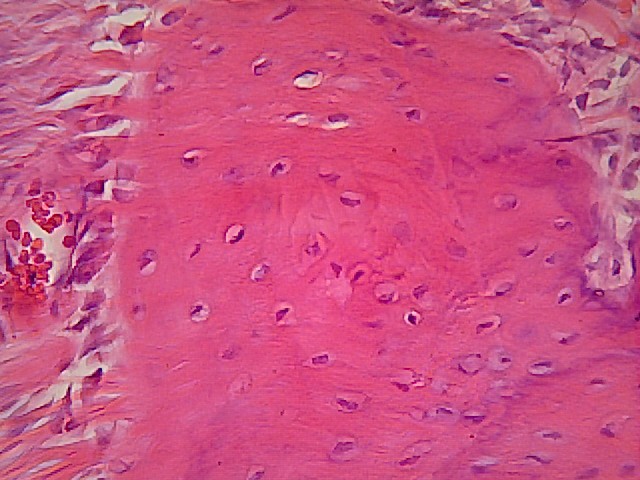
 **
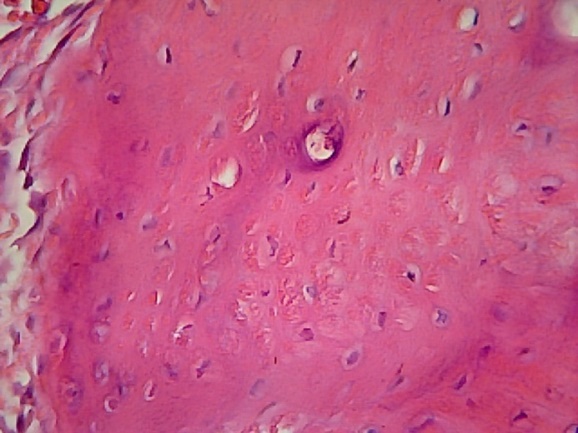
**


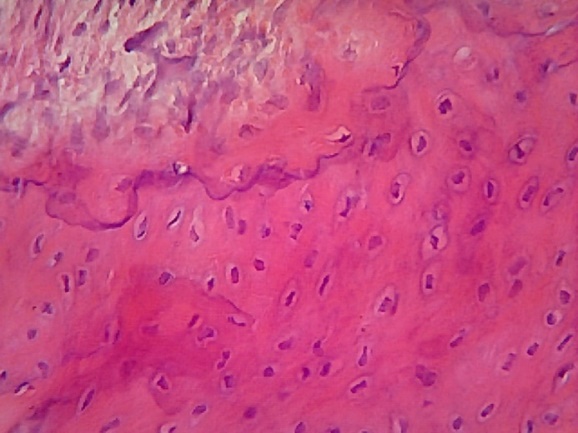
 **
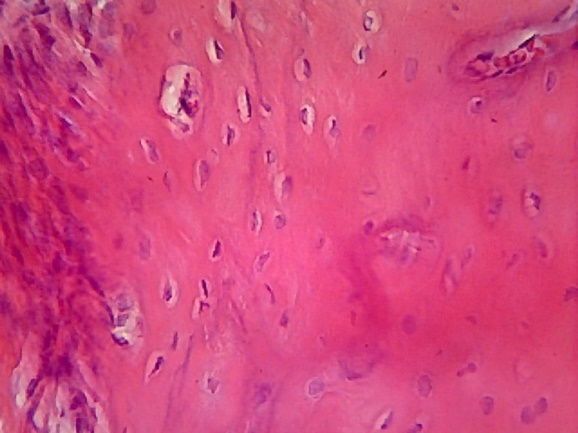
**

A2b

**
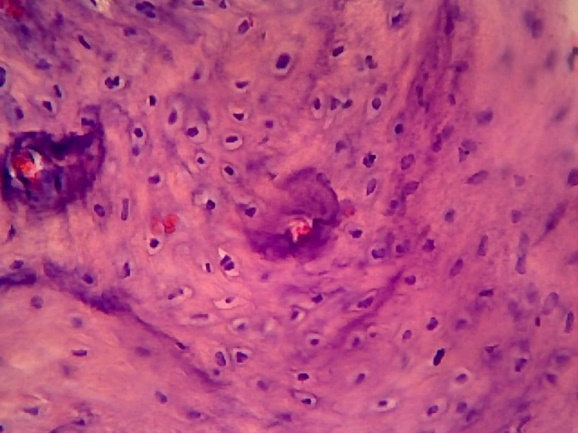
** **
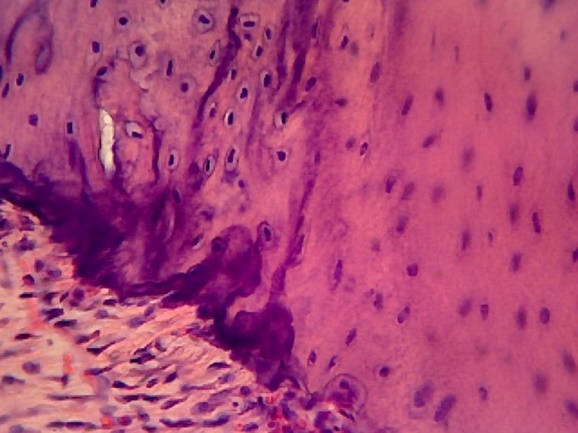
**


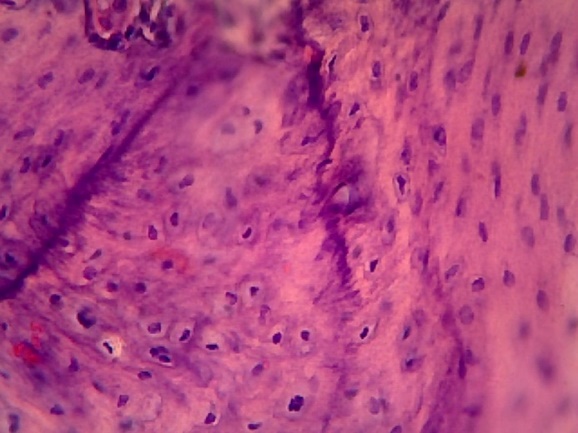

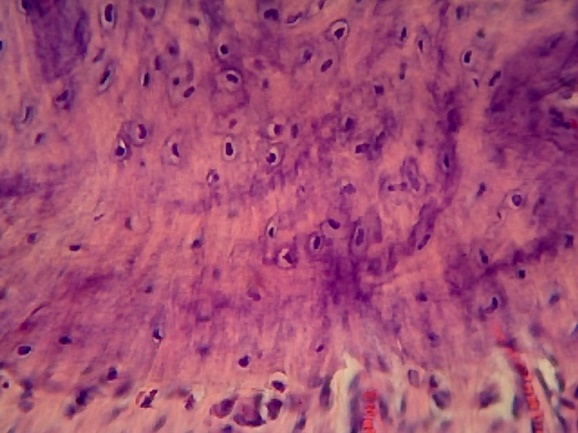


A2c


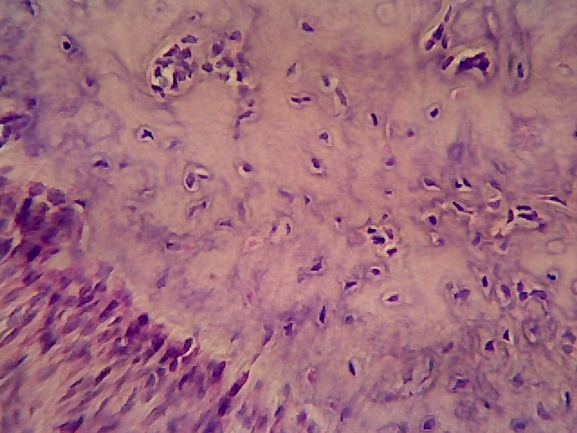

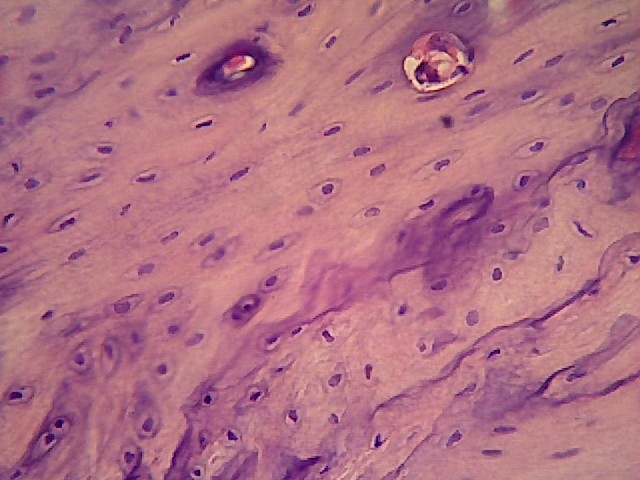

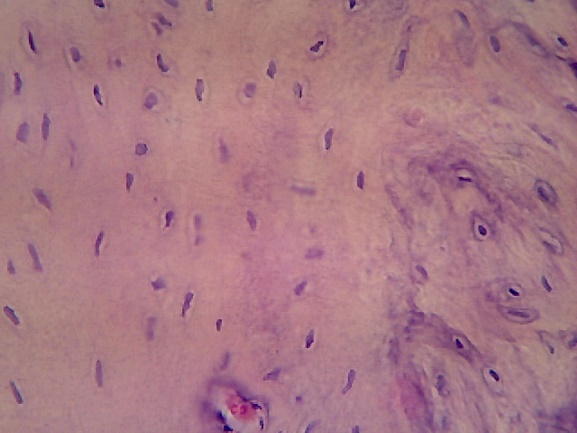

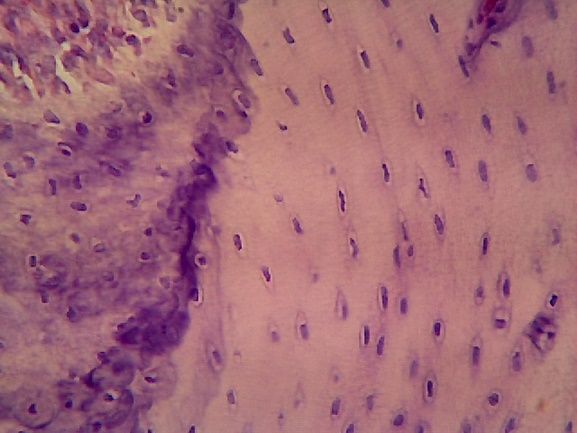


A3a

**
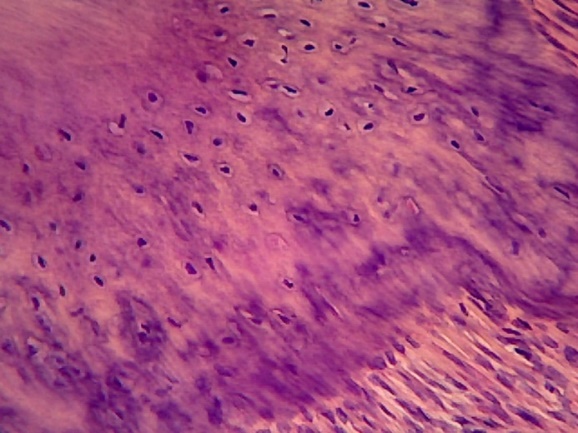
** **
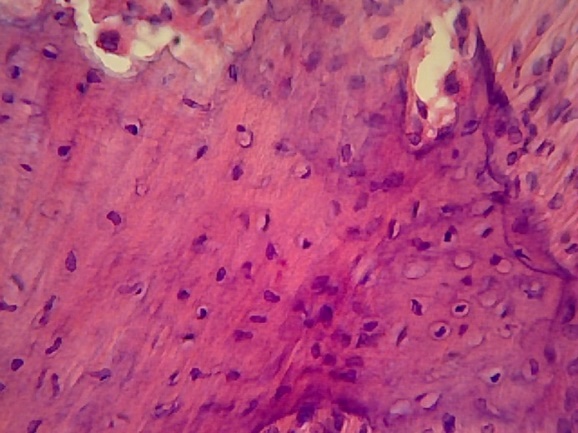
**


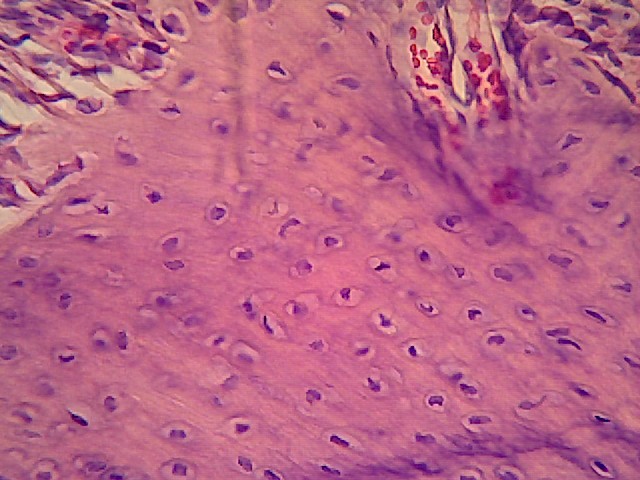

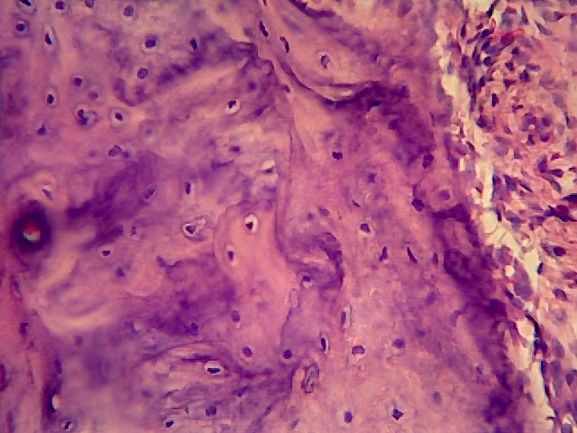


A3b


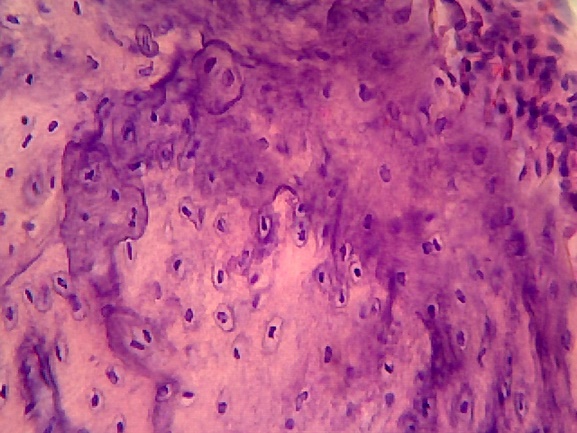

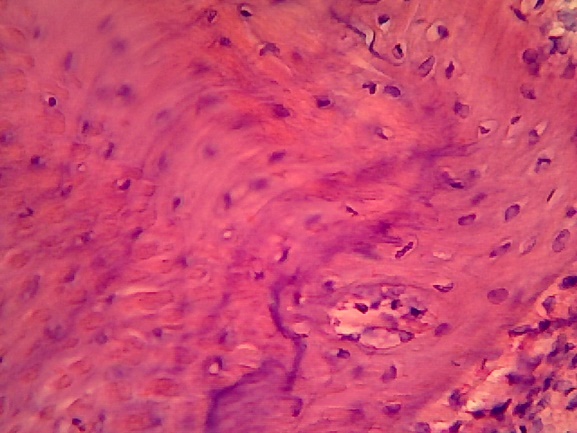


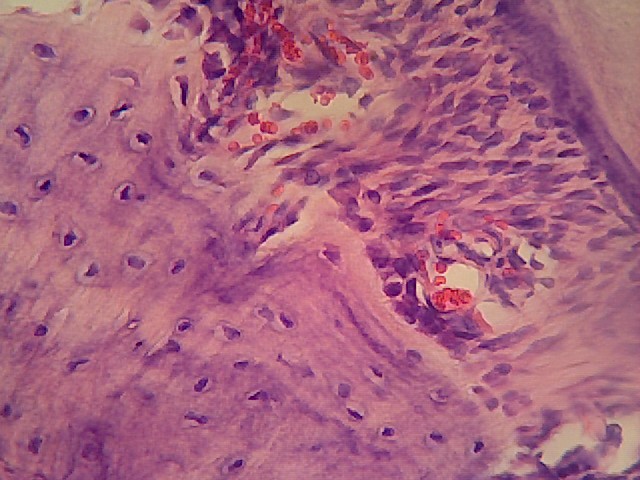

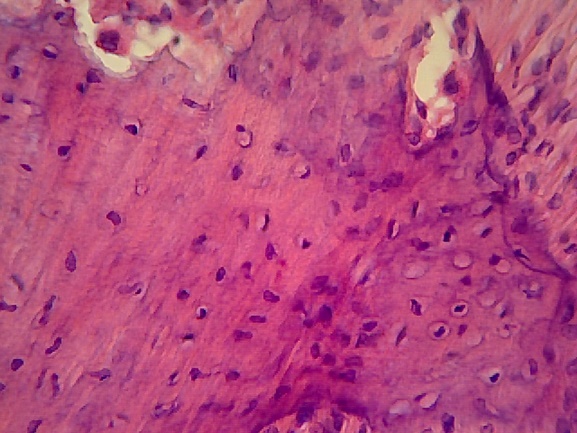


A3c


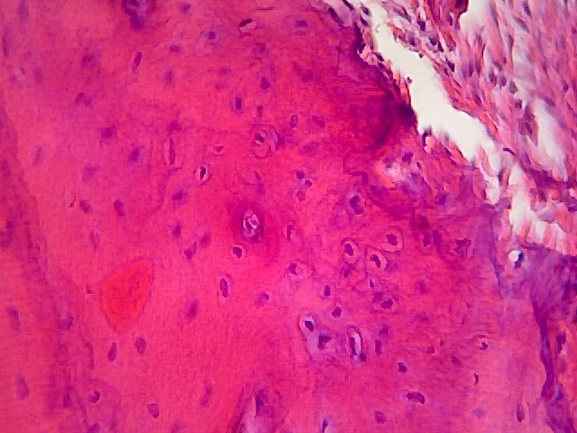

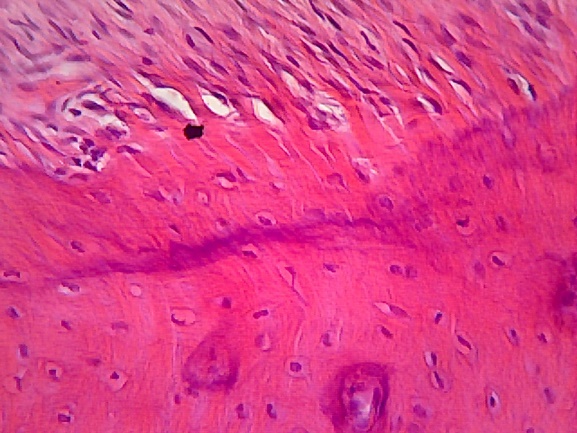

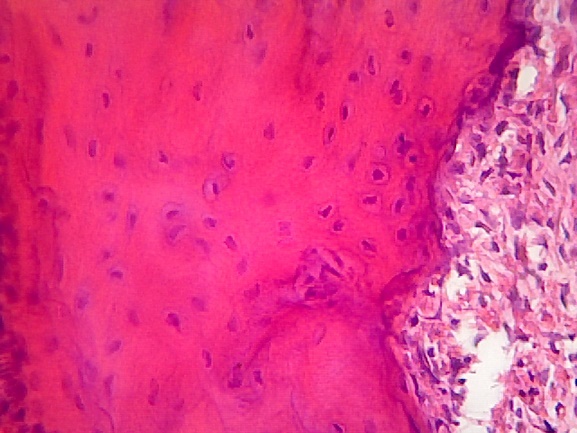

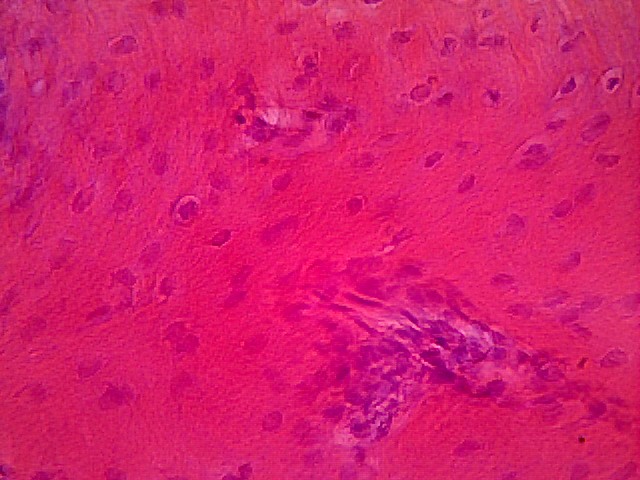


A4a

**
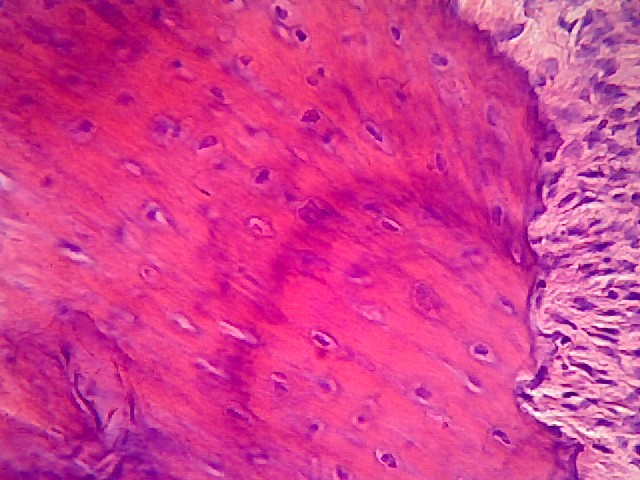
**
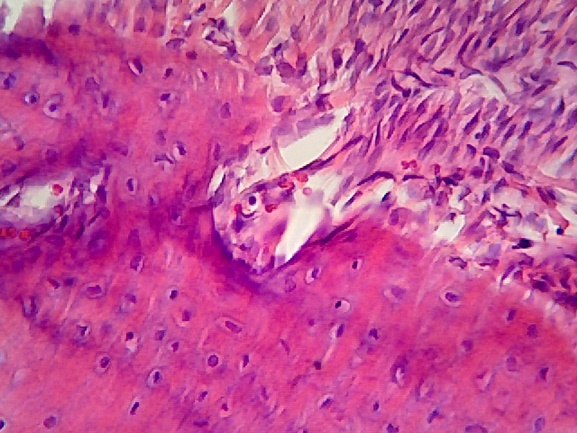


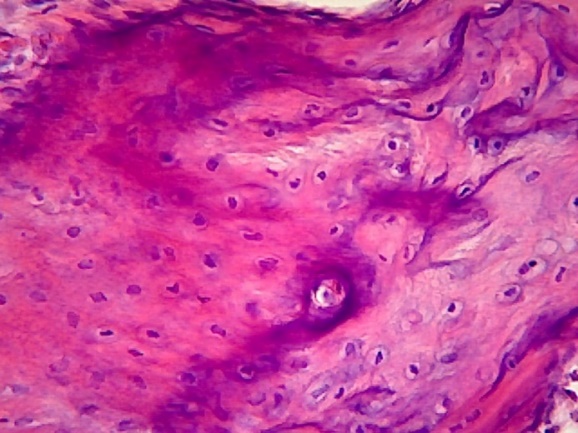

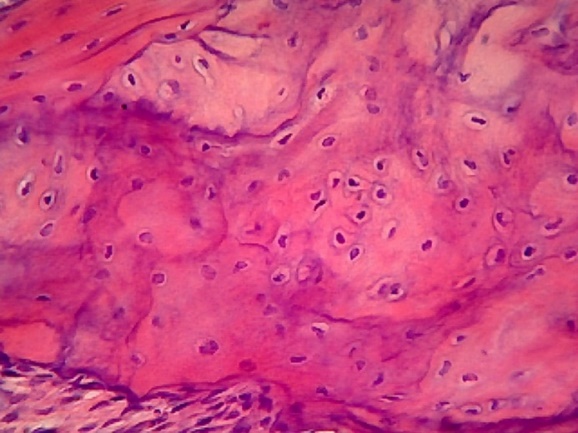


A4b

**
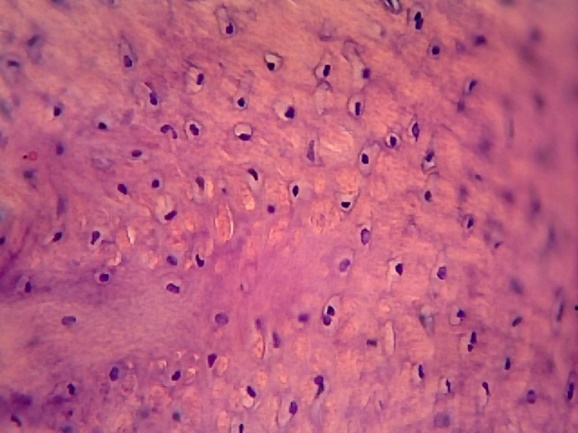
** **
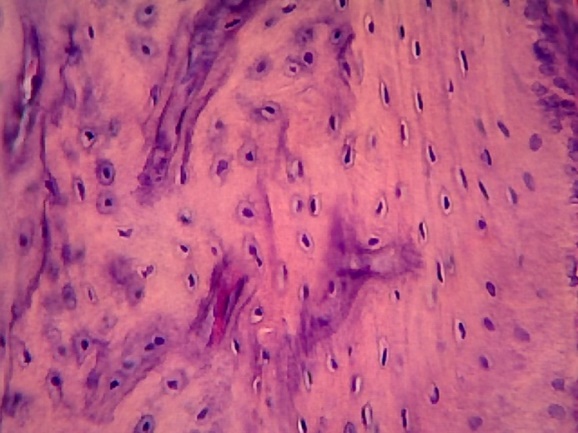
**


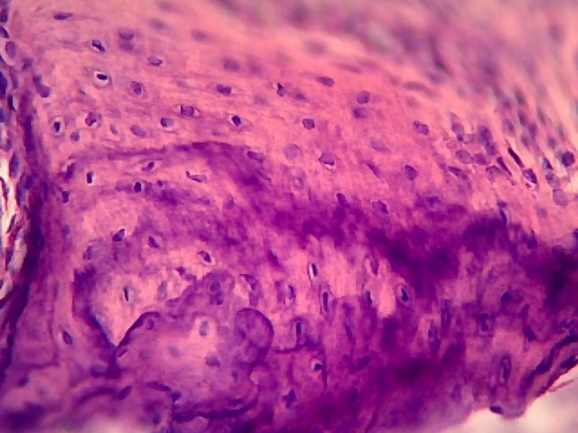

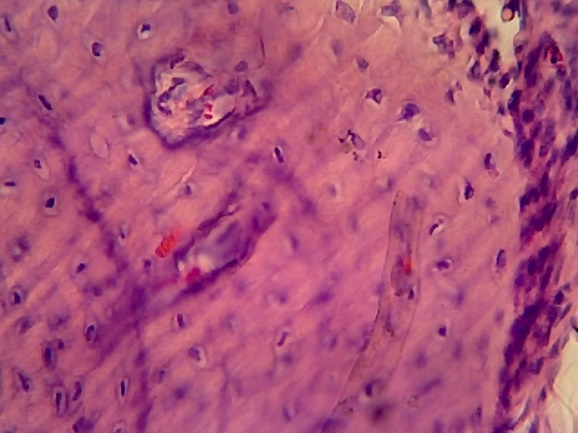


A4c

**PERIODONTITIS**

**
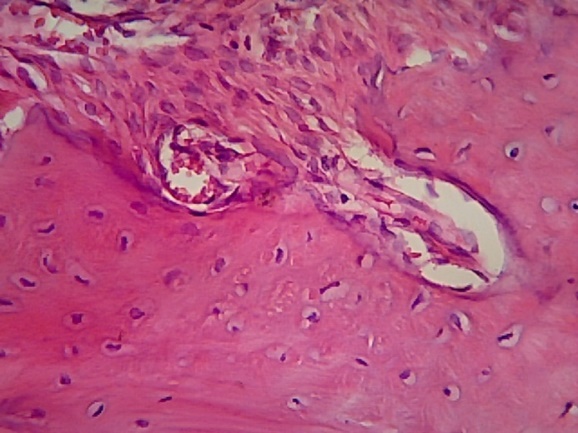

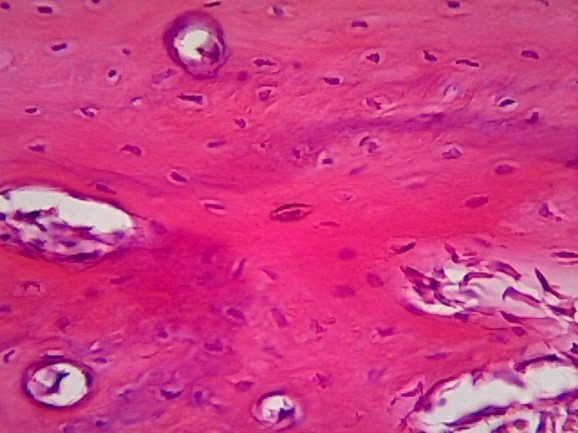
**


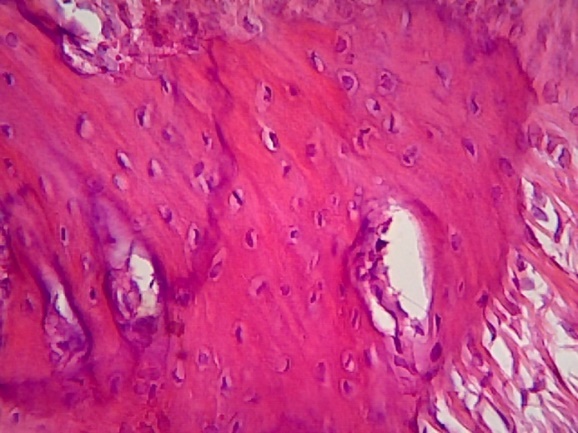

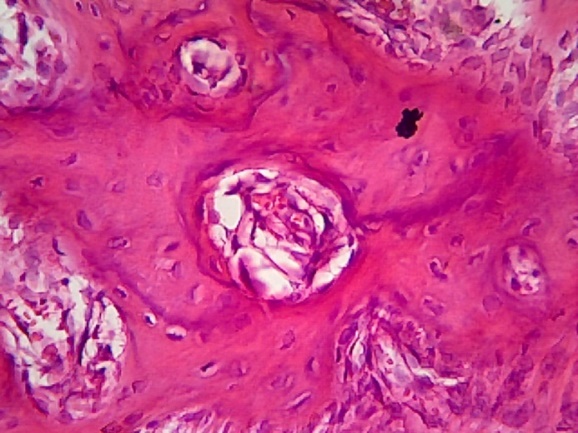


B1a

**
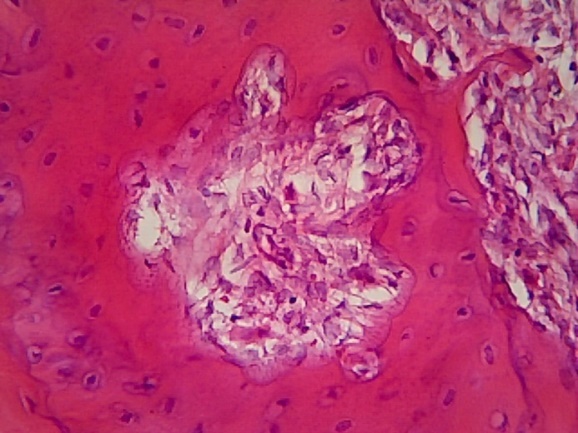
** **
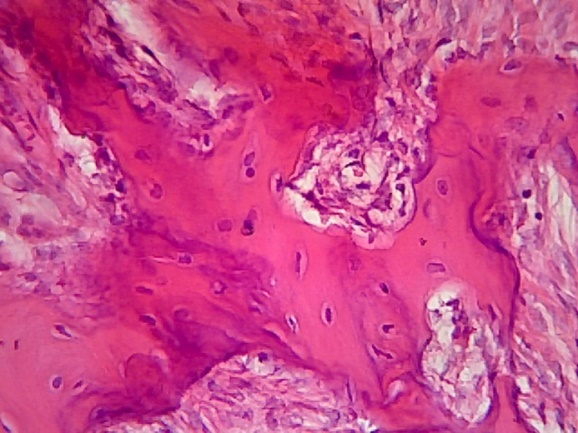
**


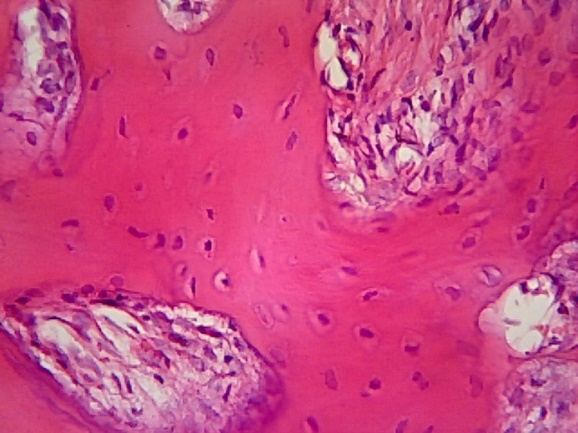

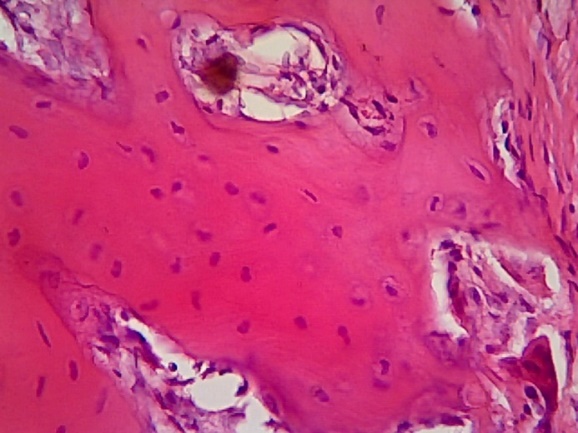


B1b

**
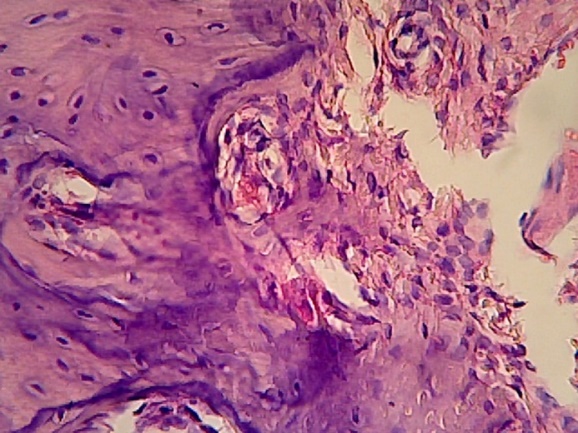
** **
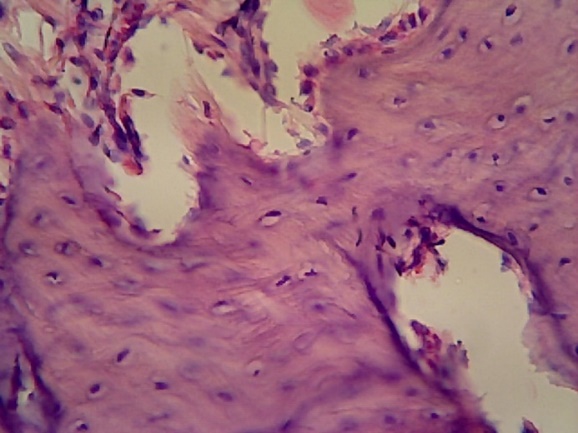
**


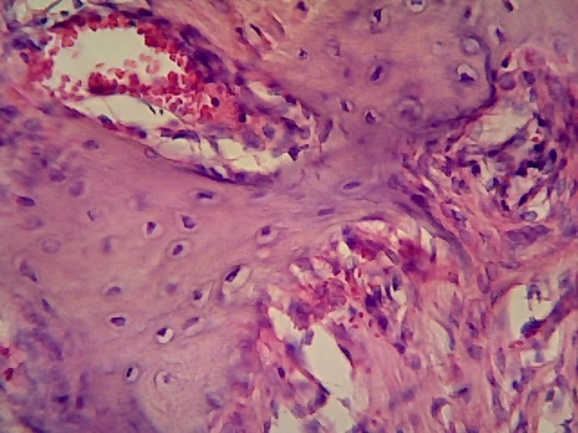

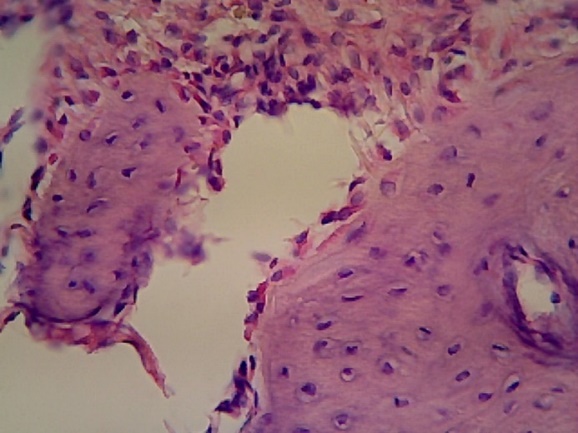


B1c

**
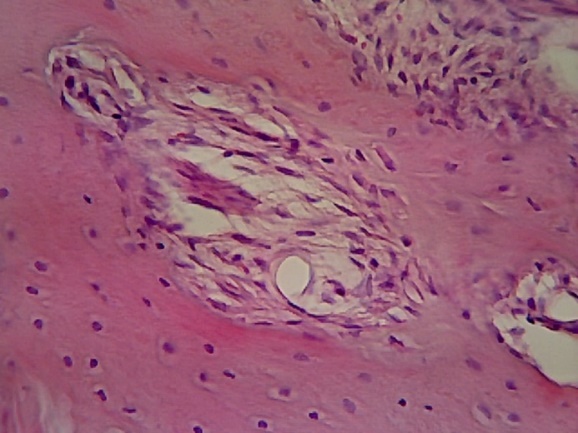
** **
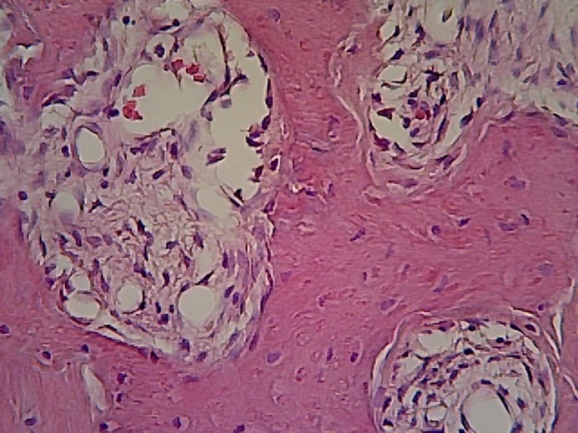
**


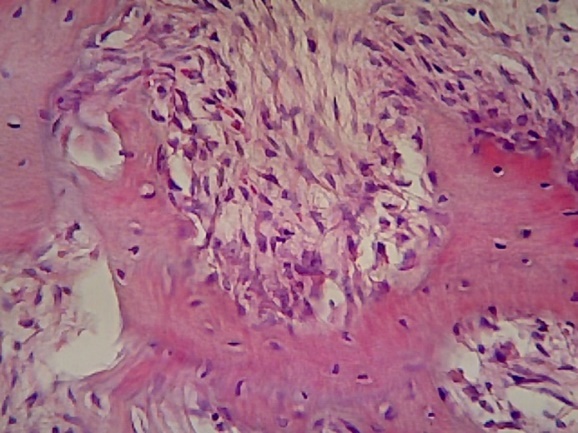

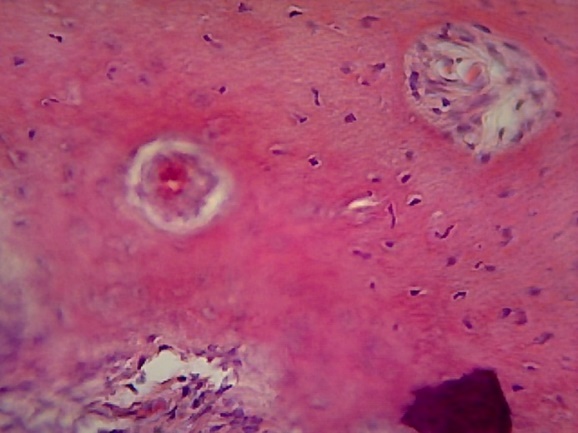


B2a

**
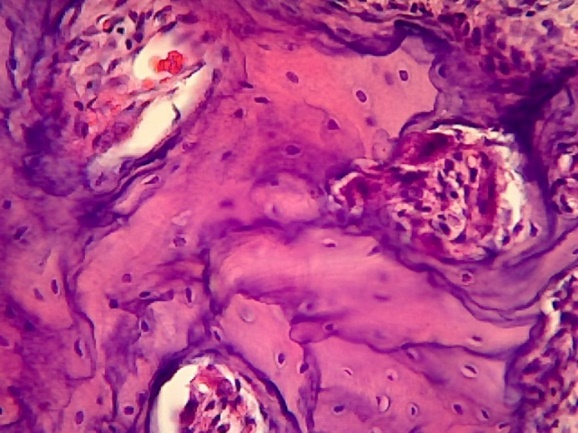
** **
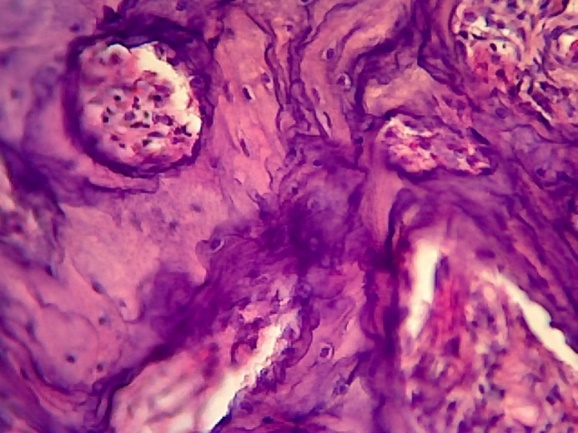
**


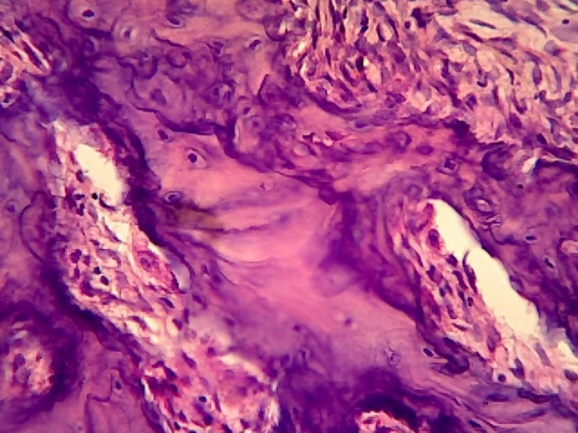

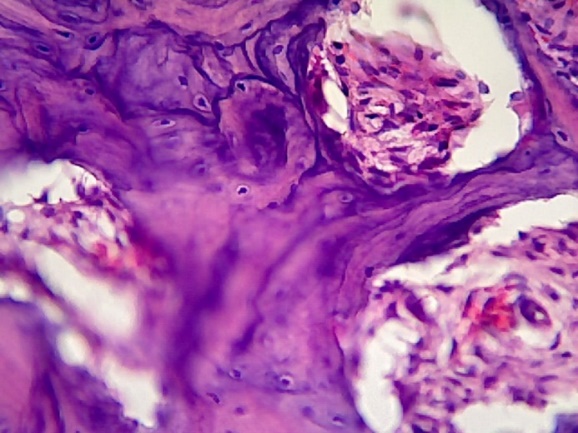


B2b

**
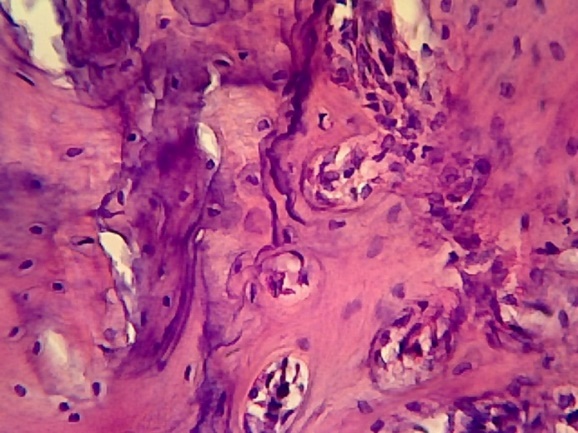
** **
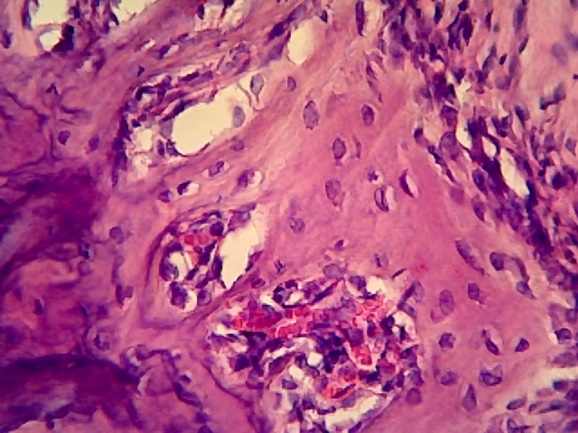
**


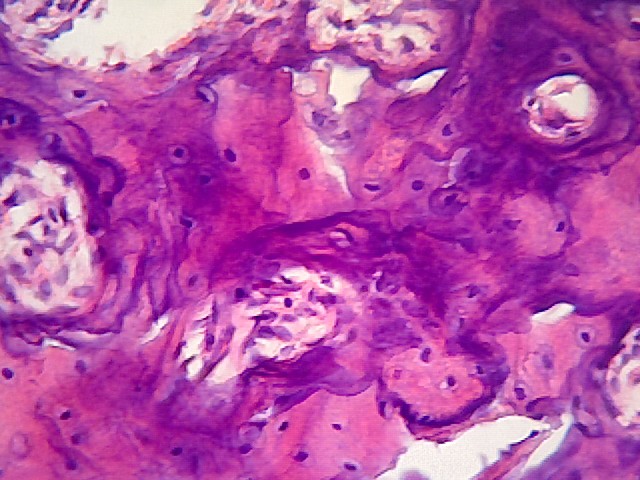

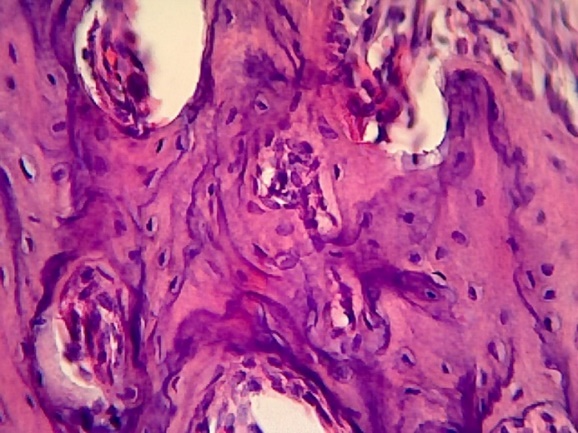


B2c

**
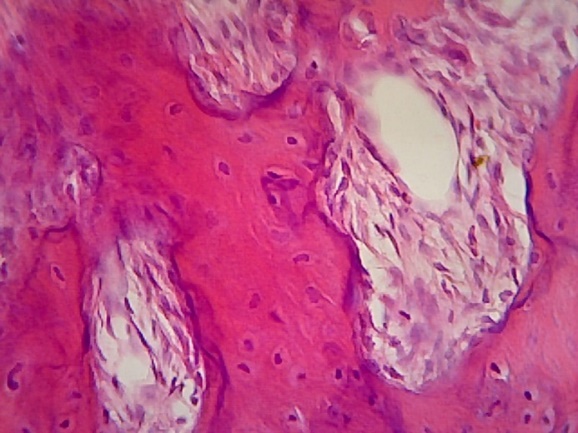
** **
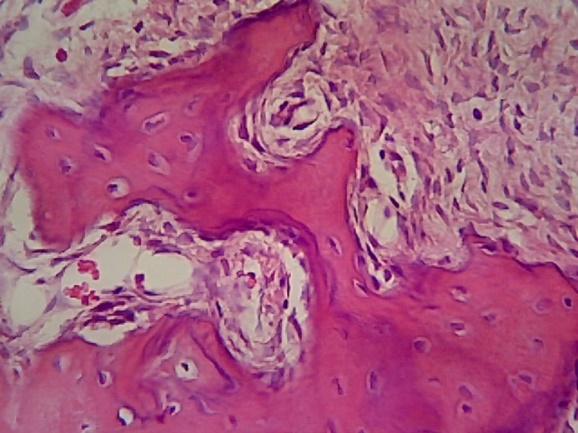
**


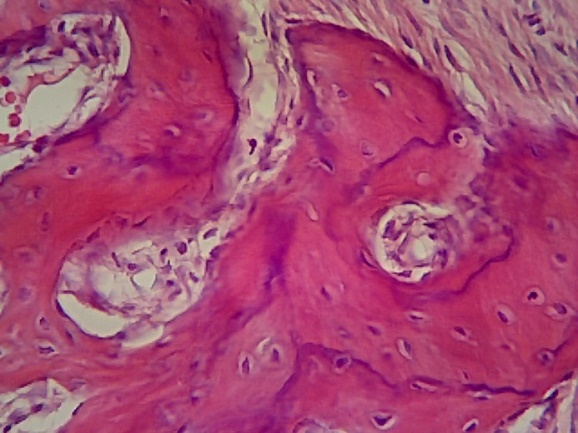

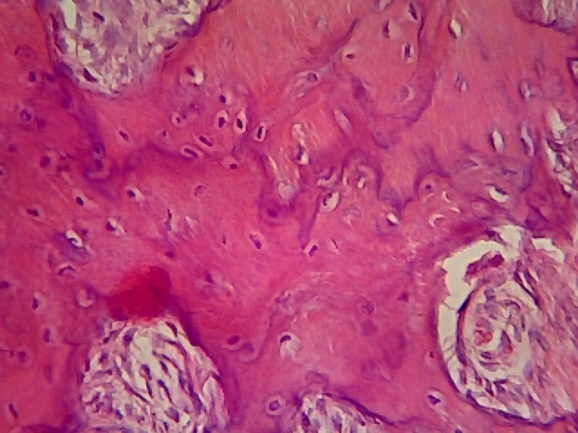


B3a

**
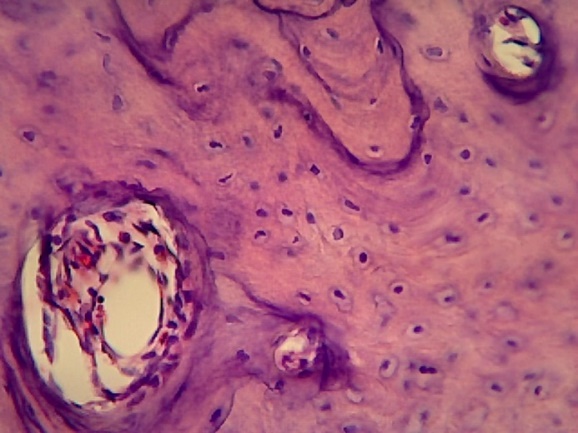
** **
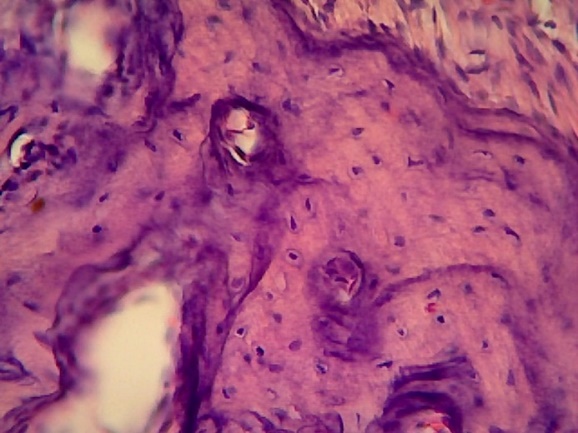
**


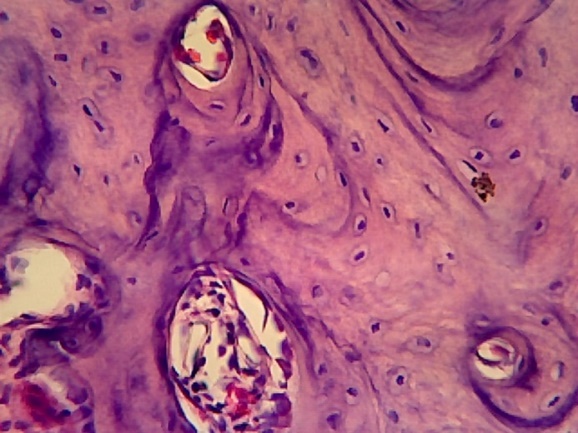

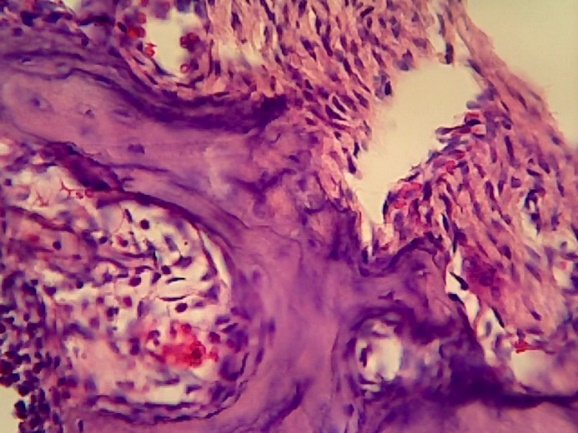


B3b

**
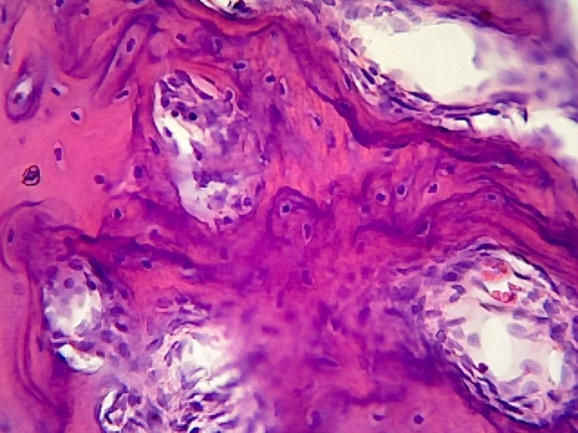
** **
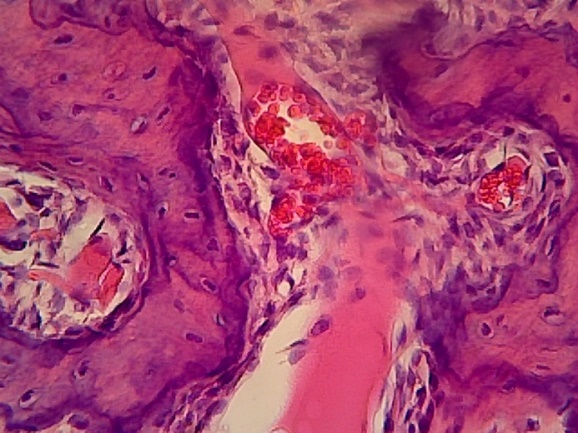
**


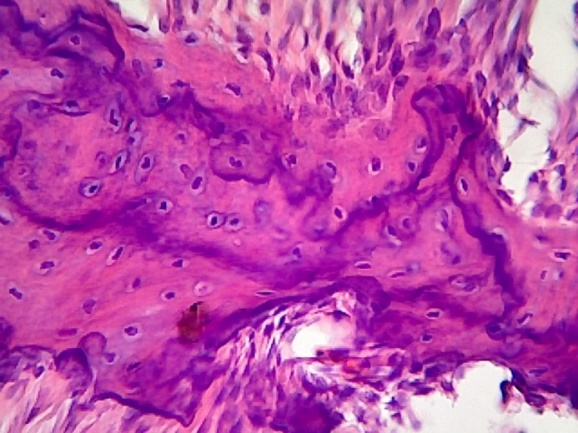

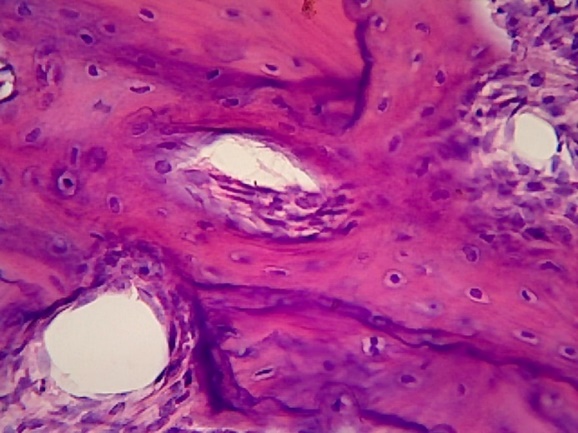


B3c

**
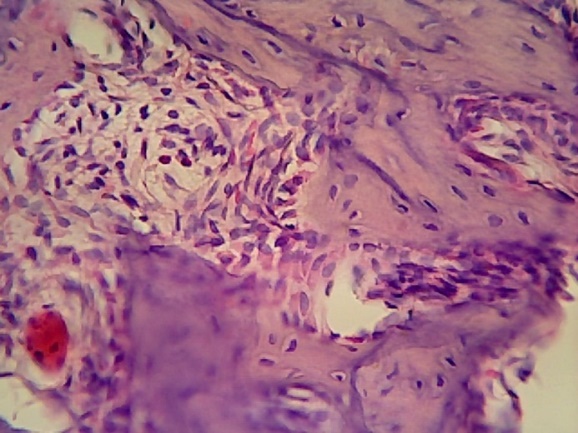
** **
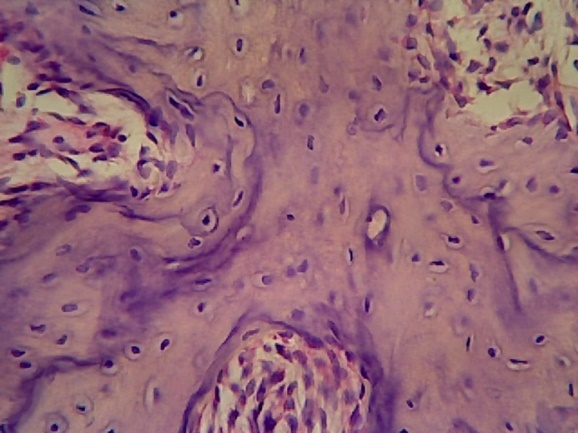
**


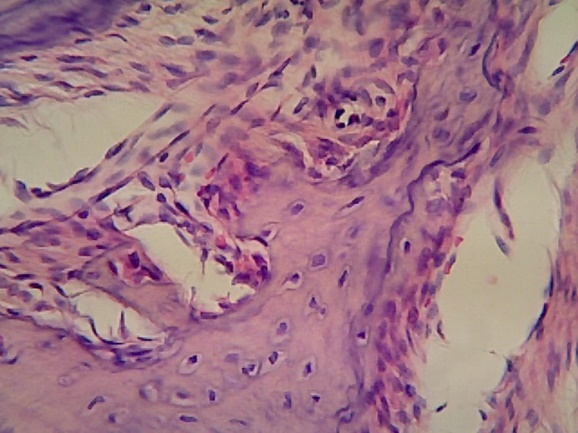

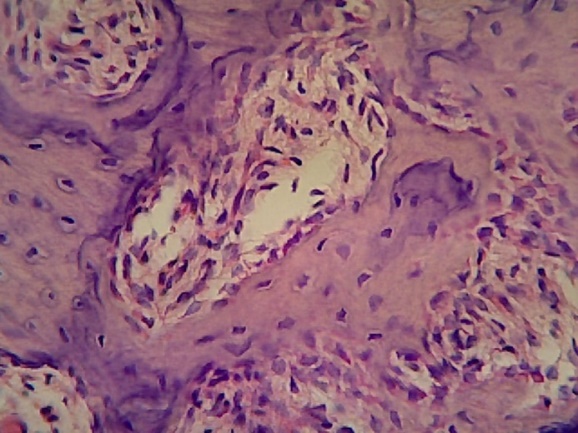


B4a

**
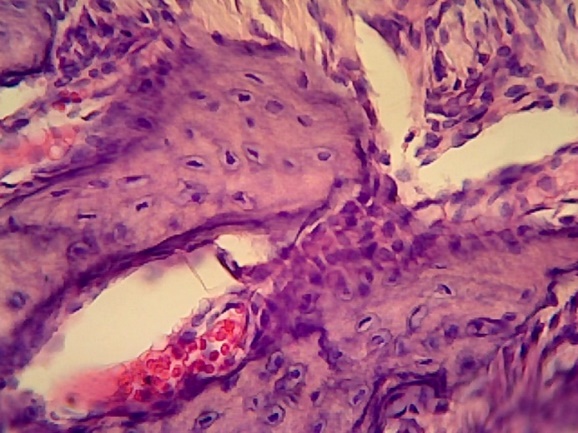
** **
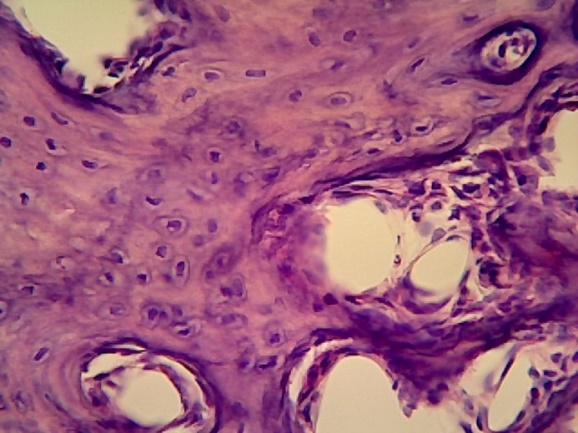
**


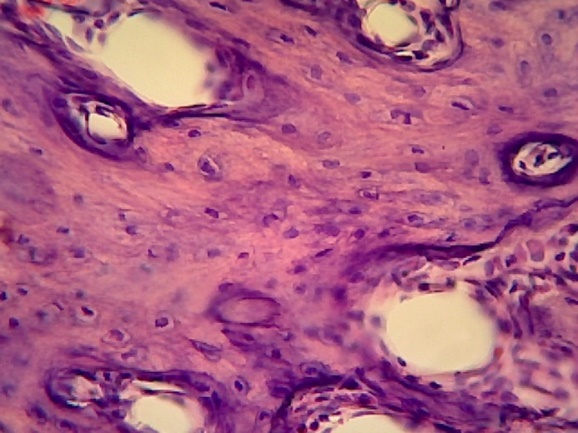

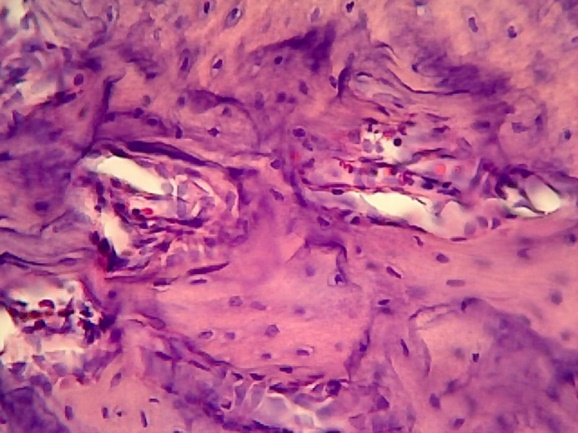


B4b

**
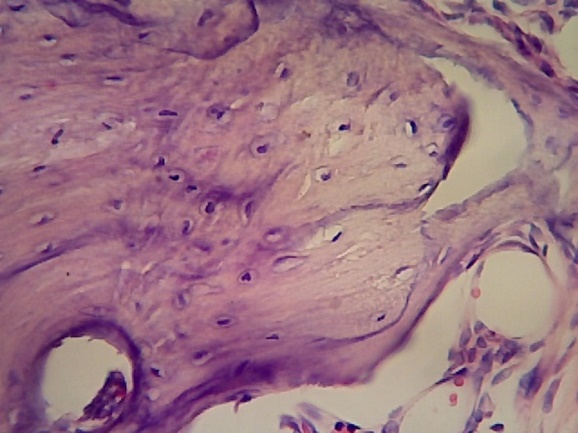
** **
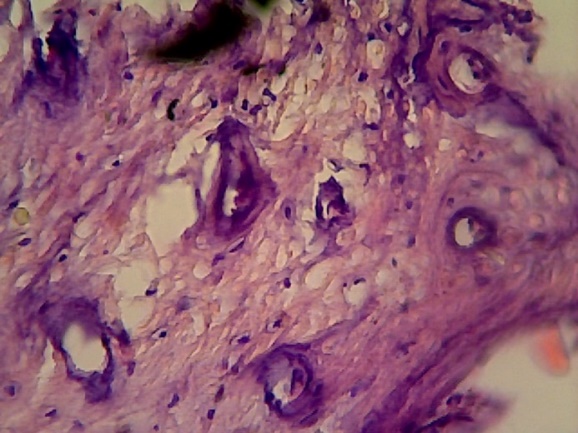
**


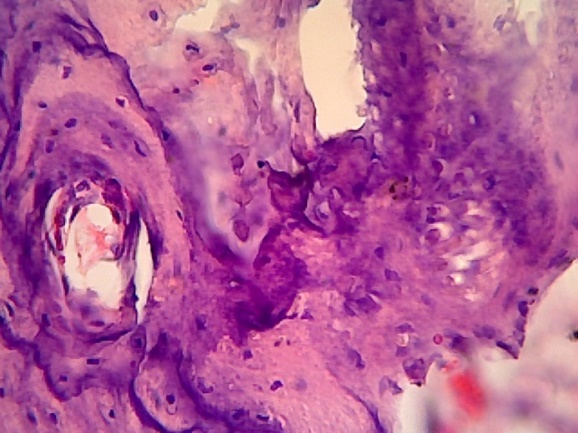

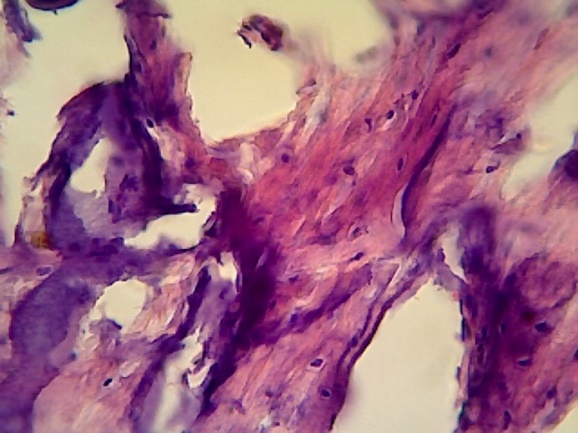


B4c

**PERIODONTITIS + LASER**

**
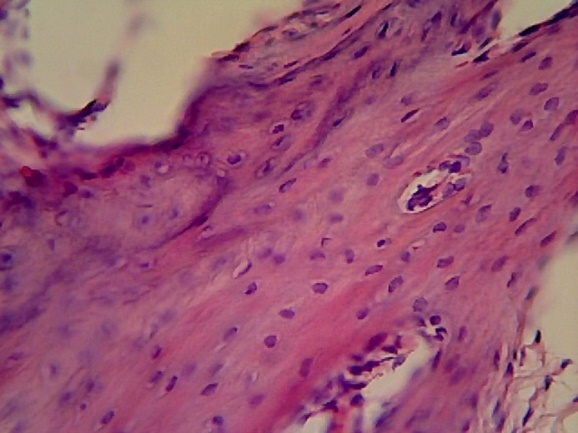
** **
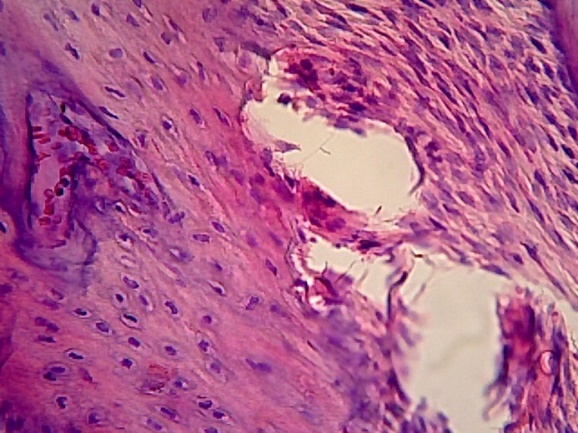
**


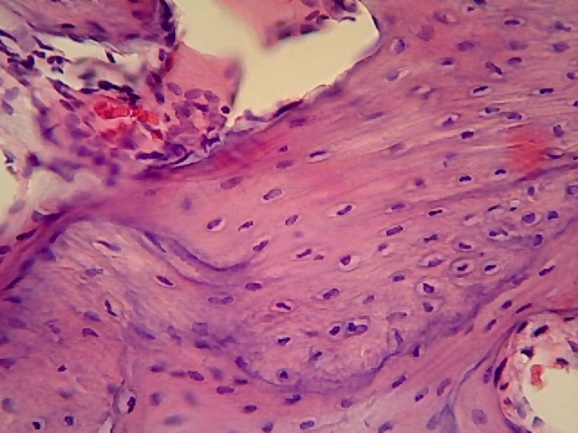

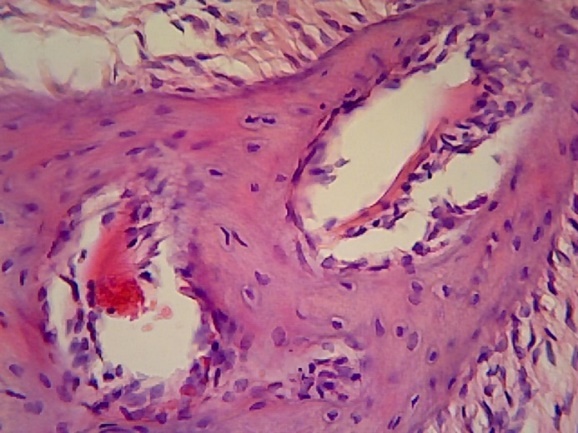


C1a

C1b

**///////////** **/**

C1c

C2a

C2b

C2c

C3a

C3b

C3c

C4a

C4b

C4c

**PERIODONTITIS + *DOXYCYLINE***

D1a

D1b

D1c

D2a

D2b

D2c

/////

D3a

D3b

D3c

D4a

///**/** **/**

D4b

D4c

**PERIODONTITIS + *DOXYCYCLINE* + LASER**

E1a

E1b

E1c

E2a

E2b

E2c

E3a

E3b

E3c

E4a

E4b

E4c

**Histomorphometric Analyses**

**1.Control Healthy Group**

| **Day**  **1** |  |
| --- | --- |
| **Day**  **3** |  |
| **Day 5** |  |
| **Day**  **7** |  |

**2.Periodontitis Group**

| **Day**  **1** |  |
| --- | --- |
| **Day**  **3** |  |
| **Day 5** |  |
| **Day**  **7** |  |

**3.Laser treatment Group**

| **Day**  **1** |  |
| --- | --- |
| **Day**  **3** |  |
| **Day 5** |  |
| **Day**  **7** |  |

**4.Doxycycline treatment Group**

| **Day**  **1** |  |
| --- | --- |
| **Day**  **3** |  |
| **Day 5** |  |
| **Day**  **7** |  |

**5.Laser-Doxycycline treatment Group**

| **Day**  **1** |  |
| --- | --- |
| **Day**  **3** |  |
| **Day 5** |  |
| **Day**  **7** |  |

**macrophages**

**lymphocytes**

**Fibroblast**
