# Supplementary material for: Combination effect of laser diode for photodynamic therapy with doxycycline on a wistar rat model of periodontitis
Source: BMC Oral Health. 2021 Feb 19;21:80. doi: 10.1186/s12903-021-01435-0 (PMC7893773; doi:10.1186/s12903-021-01435-0)
Supplement: Supplementary file 3 — Additional file 3. Meta data-3 Statistical analysis. [file 12903_2021_1435_MOESM3_ESM.docx]

**Statistical Analysis**

**Immunocompetent cell statistical tests**

**Data normality test.**

**One-Sample Kolmogorov-Smirnov Test**

|  | | Makrofag | Limfosit | Fibroblas |
| --- | --- | --- | --- | --- |
| N | | 60 | 60 | 60 |
| Normal Parameters(a,b) | Mean | 5,57 | 6,98 | 27,05 |
|  | Std. Deviation | 2,417 | 2,671 | 5,832 |
| Most Extreme Differences | Absolute | ,146 | ,127 | ,084 |
|  | Positive | ,146 | ,127 | ,084 |
|  | Negative | -,077 | -,068 | -,081 |
| Kolmogorov-Smirnov Z | | 1,127 | ,983 | ,647 |
| Asymp. Sig. (2-tailed) | | ,157 | ,288 | ,796 |

a Test distribution is Normal.

b Calculated from data.

**Data Variance Test**

**Test of Homogeneity of Variances**

|  | Levene Statistic | df1 | df2 | Sig. |
| --- | --- | --- | --- | --- |
| Makrofag | 3,216 | 19 | 40 | ,001 |
| Limfosit | 3,960 | 19 | 40 | ,000 |
| Fibroblas | 2,267 | 19 | 40 | ,015 |

***Kruskal Wallis test***

**Ranks**

| COMBINATION | | N | Mean Rank |
| --- | --- | --- | --- |
| Macrophages | Day-1 normal (S1) | 3 | 27,67 |
|  | Day-3 normal (S2) | 3 | 18,50 |
|  | Day-5 normal (S3) | 3 | 27,83 |
|  | Day-7 normal (S4) | 3 | 27,67 |
|  | Day-1 Periodontitis (P1) | 3 | 53,50 |
|  | Day-3 Periodontitis (P2) | 3 | 49,17 |
|  | Day-5 Periodontitis (P3) | 3 | 55,83 |
|  | Day-7 Periodontitis (P4) | 3 | 51,67 |
|  | Day-1 Laser treatment (PL1) | 3 | 30,33 |
|  | Day-3 Laser treatment (PL3) | 3 | 25,00 |
|  | Day-5 Laser treatment (PL5) | 3 | 33,83 |
|  | Day-7 Laser treatment (PL7) | 3 | 23,33 |
|  | Day-1 Doxycyclin treatment (PD1) | 3 | 38,33 |
|  | Day-3 Doxycyclin treatment (PD3) | 3 | 37,00 |
|  | Day-5 Doxycyclin treatment (PD5) | 3 | 43,00 |
|  | Day-7 Doxycyclin treatment (PD7) | 3 | 32,33 |
|  | Day-1 Laser-Doxycyclin treatment (PLD1) | 3 | 13,50 |
|  | Day-3 Laser-Doxycyclin treatment (PLD3) | 3 | 9,50 |
|  | Day-5 Laser-Doxycyclin treatment (PLD5) | 3 | 9,50 |
|  | Day-7 Laser-Doxycyclin treatment (PLD7) | 3 | 2,50 |
|  | Total | 60 |  |
| Limfosit | Day-1 normal (S1) | 3 | 6,83 |
|  | Day-3 normal (S2) | 3 | 2,50 |
|  | Day-5 normal (S3) | 3 | 14,33 |
|  | Day-7 normal (S4) | 3 | 9,00 |
|  | Day-1 Periodontitis (P1) | 3 | 22,17 |
|  | Day-3 Periodontitis (P2) | 3 | 19,67 |
|  | Day-5 Periodontitis (P3) | 3 | 22,33 |
|  | Day-7 Periodontitis (P4) | 3 | 17,00 |
|  | Day-1 Laser treatment (PL1) | 3 | 33,67 |
|  | Day-3 Laser treatment (PL3) | 3 | 47,83 |
|  | Day-5 Laser treatment (PL5) | 3 | 42,33 |
|  | Day-7 Laser treatment (PL7) | 3 | 43,67 |
|  | Day-1 Doxycyclin treatment (PD1) | 3 | 19,67 |
|  | Day-3 Doxycyclin treatment (PD3) | 3 | 42,33 |
|  | Day-5 Doxycyclin treatment (PD5) | 3 | 35,00 |
|  | Day-7 Doxycyclin treatment (PD7) | 3 | 30,00 |
|  | Day-1 Laser-Doxycyclin treatment (PLD1) | 3 | 45,17 |
|  | Day-3 Laser-Doxycyclin treatment (PLD3) | 3 | 53,00 |
|  | Day-5 Laser-Doxycyclin treatment (PLD5) | 3 | 52,83 |
|  | Day-7 Laser-Doxycyclin treatment (PLD7) | 3 | 50,67 |
|  | Total | 60 |  |
| Fibroblasts | Day-1 normal (S1) | 3 | 47,00 |
|  | Day-3 normal (S2) | 3 | 46,50 |
|  | Day-5 normal (S3) | 3 | 52,83 |
|  | Day-7 normal (S4) | 3 | 52,50 |
|  | Day-1 Periodontitis (P1) | 3 | 6,00 |
|  | Day-3 Periodontitis (P2) | 3 | 5,17 |
|  | Day-5 Periodontitis (P3) | 3 | 7,83 |
|  | Day-7 Periodontitis (P4) | 3 | 12,83 |
|  | Day-1 Laser treatment (PL1) | 3 | 25,00 |
|  | Day-3 Laser treatment (PL3) | 3 | 26,50 |
|  | Day-5 Laser treatment (PL5) | 3 | 33,00 |
|  | Day-7 Laser treatment (PL7) | 3 | 33,33 |
|  | Day-1 Doxycyclin treatment (PD1) | 3 | 14,67 |
|  | Day-3 Doxycyclin treatment (PD3) | 3 | 11,50 |
|  | Day-5 Doxycyclin treatment (PD5) | 3 | 20,50 |
|  | Day-7 Doxycyclin treatment (PD7) | 3 | 33,33 |
|  | Day-1 Laser-Doxycyclin treatment (PLD1) | 3 | 40,50 |
|  | Day-3 Laser-Doxycyclin treatment (PLD3) | 3 | 42,67 |
|  | Day-5 Laser-Doxycyclin treatment (PLD5) | 3 | 51,67 |
|  | Day-7 Laser-Doxycyclin treatment (PLD7) | 3 | 46,67 |
|  | Total | 60 |  |

**Test Statistics(a,b)**

|  | Makrofag | Limfosit | Fibroblas |
| --- | --- | --- | --- |
| Chi-Square | 44,698 | 50,194 | 52,516 |
| df | 19 | 19 | 19 |
| Asymp. Sig. | ,001 | ,000 | ,000 |

a Kruskal Wallis Test

b Grouping Variable: Combination

***Mann Whitney post hoc test***

| **MACROPHAGES** | | | | | | | | | | | | | | | | | | | |
| --- | --- | --- | --- | --- | --- | --- | --- | --- | --- | --- | --- | --- | --- | --- | --- | --- | --- | --- | --- |
|  |  |  |  |  |  |  |  |  |  |  |  |  |  |  |  |  |  |  |  |
| **S1** | **S3** | **S5** | **S7** | **P1** | **P3** | **P5** | **P7** | **PL1** | **PL3** | **PL5** | **PL7** | **PD1** | **PD3** | **PD5** | **PD7** | **PLD1** | **PLD3** | **PLD5** | **PLD7** |
| S1 | T | T | T | S | S | S | S | T | T | T | T | T | T | T | T | T | S | S | S |
| S3 |  | T | T | S | S | S | S | T | T | T | T | T | S | S | T | T | T | T | S |
| S5 |  |  | T | S | S | S | S | T | T | T | T | T | T | T | T | T | T | T | S |
| S7 |  |  |  | S | S | S | S | T | T | T | T | T | T | T | T | T | S | S | S |
| P1 |  |  |  |  | T | T | T | S | S | T | T | T | S | T | T | S | S | S | S |
| P3 |  |  |  |  |  | T | T | S | S | T | T | T | S | T | T | S | S | S | S |
| P5 |  |  |  |  |  |  | T | S | S | T | T | T | S | T | T | S | S | S | S |
| P7 |  |  |  |  |  |  |  | S | S | T | T | T | S | T | T | S | S | S | S |
| PL1 |  |  |  |  |  |  |  |  | T | T | T | T | T | T | T | S | S | S | S |
| PL3 |  |  |  |  |  |  |  |  |  | T | T | T | T | T | T | T | S | S | S |
| PL5 |  |  |  |  |  |  |  |  |  |  | T | T | T | T | T | T | S | S | S |
| PL7 |  |  |  |  |  |  |  |  |  |  |  | T | T | T | T | T | T | T | S |
| PD1 |  |  |  |  |  |  |  |  |  |  |  |  | T | T | T | S | S | S | S |
| PD3 |  |  |  |  |  |  |  |  |  |  |  |  |  | T | S | S | S | S | S |
| PD5 |  |  |  |  |  |  |  |  |  |  |  |  |  |  | T | S | S | S | S |
| PD7 |  |  |  |  |  |  |  |  |  |  |  |  |  |  |  | T | S | S | S |
| PLD1 |  |  |  |  |  |  |  |  |  |  |  |  |  |  |  |  | T | T | T |
| PLD3 |  |  |  |  |  |  |  |  |  |  |  |  |  |  |  |  |  | T | S |
| PLD5 |  |  |  |  |  |  |  |  |  |  |  |  |  |  |  |  |  |  | S |
| PLD7 |  |  |  |  |  |  |  |  |  |  |  |  |  |  |  |  |  |  |  |

| **LIMFOSIT** | | | | | | | | | | | | | | | | | | | |
| --- | --- | --- | --- | --- | --- | --- | --- | --- | --- | --- | --- | --- | --- | --- | --- | --- | --- | --- | --- |
|  |  |  |  |  |  |  |  |  |  |  |  |  |  |  |  |  |  |  |  |
| **S1** | **S3** | **S5** | **S7** | **P1** | **P3** | **P5** | **P7** | **PL1** | **PL3** | **PL5** | **PL7** | **PD1** | **PD3** | **PD5** | **PD7** | **PLD1** | **PLD3** | **PLD5** | **PLD7** |
| **S1** | T | T | T | T | S | S | T | S | S | S | S | T | S | S | S | S | S | S | S |
| **S3** |  | S | S | S | S | S | S | S | S | S | S | S | S | S | S | S | S | S | S |
| **S5** |  |  | T | T | T | T | T | T | S | S | S | T | S | S | S | S | S | S | S |
| **S7** |  |  |  | T | S | S | T | S | S | S | S | T | S | S | S | S | S | S | S |
| **P1** |  |  |  |  | T | T | T | T | T | S | T | T | S | T | T | S | S | S | S |
| **P3** |  |  |  |  |  | T | T | T | S | S | S | T | S | S | T | S | S | S | S |
| **P5** |  |  |  |  |  |  | T | T | S | S | S | T | S | S | T | S | S | S | S |
| **P7** |  |  |  |  |  |  |  | T | S | S | S | S | S | S | T | S | S | S | S |
| **PL1** |  |  |  |  |  |  |  |  | T | T | T | T | T | T | T | T | T | T | T |
| **PL3** |  |  |  |  |  |  |  |  |  | T | T | S | T | T | T | T | T | T | T |
| **PL5** |  |  |  |  |  |  |  |  |  |  | T | S | T | T | T | T | T | S | T |
| **PL7** |  |  |  |  |  |  |  |  |  |  |  | S | T | T | T | T | T | T | T |
| **PD1** |  |  |  |  |  |  |  |  |  |  |  |  | S | S | T | S | S | S | S |
| **PD3** |  |  |  |  |  |  |  |  |  |  |  |  |  | T | T | T | T | S | T |
| **PD5** |  |  |  |  |  |  |  |  |  |  |  |  |  |  | T | T | T | S | S |
| **PD7** |  |  |  |  |  |  |  |  |  |  |  |  |  |  |  | T | T | S | S |
| **PLD1** |  |  |  |  |  |  |  |  |  |  |  |  |  |  |  |  | T | T | T |
| **PLD3** |  |  |  |  |  |  |  |  |  |  |  |  |  |  |  |  |  | T | T |
| **PLD5** |  |  |  |  |  |  |  |  |  |  |  |  |  |  |  |  |  |  | T |
| **PLD7** |  |  |  |  |  |  |  |  |  |  |  |  |  |  |  |  |  |  |  |

| **FIBROBLAS** | | | | | | | | | | | | | | | | | | | |
| --- | --- | --- | --- | --- | --- | --- | --- | --- | --- | --- | --- | --- | --- | --- | --- | --- | --- | --- | --- |
|  |  |  |  |  |  |  |  |  |  |  |  |  |  |  |  |  |  |  |  |
| **S1** | **S3** | **S5** | **S7** | **P1** | **P3** | **P5** | **P7** | **PL1** | **PL3** | **PL5** | **PL7** | **PD1** | **PD3** | **PD5** | **PD7** | **PLD1** | **PLD3** | **PLD5** | **PLD7** |
| **S1** | T | T | T | S | S | S | S | S | S | S | T | S | S | S | T | T | T | T | T |
| **S3** |  | T | T | S | S | S | S | S | S | T | T | S | S | S | T | T | T | T | T |
| **S5** |  |  | T | S | S | S | S | S | S | T | T | S | S | S | T | T | T | T | T |
| **S7** |  |  |  | S | S | S | S | S | S | T | T | S | S | S | T | T | T | T | T |
| **P1** |  |  |  |  | T | T | T | S | S | S | S | S | S | S | S | S | S | S | S |
| **P3** |  |  |  |  |  | T | T | S | S | S | S | T | T | S | S | S | S | S | S |
| **P5** |  |  |  |  |  |  | T | S | S | S | S | T | T | S | S | S | S | S | S |
| **P7** |  |  |  |  |  |  |  | T | S | S | S | T | T | T | S | S | S | S | S |
| **PL1** |  |  |  |  |  |  |  |  | T | S | T | T | S | T | T | T | S | S | S |
| **PL3** |  |  |  |  |  |  |  |  |  | S | T | S | S | T | T | T | S | S | S |
| **PL5** |  |  |  |  |  |  |  |  |  |  | T | S | S | S | T | T | S | T | T |
| **PL7** |  |  |  |  |  |  |  |  |  |  |  | S | S | T | T | T | T | T | T |
| **PD1** |  |  |  |  |  |  |  |  |  |  |  |  | T | T | S | S | S | S | S |
| **PD3** |  |  |  |  |  |  |  |  |  |  |  |  |  | S | S | S | S | S | T |
| **PD5** |  |  |  |  |  |  |  |  |  |  |  |  |  |  | T | S | S | S | S |
| **PD7** |  |  |  |  |  |  |  |  |  |  |  |  |  |  |  | T | T | T | T |
| **PLD1** |  |  |  |  |  |  |  |  |  |  |  |  |  |  |  |  | T | T | T |
| **PLD3** |  |  |  |  |  |  |  |  |  |  |  |  |  |  |  |  |  | T | T |
| **PLD5** |  |  |  |  |  |  |  |  |  |  |  |  |  |  |  |  |  |  | T |
| **PLD7** |  |  |  |  |  |  |  |  |  |  |  |  |  |  |  |  |  |  |  |

**CEJ-AV statistical test**

**Test data normality**

| **One-Sample Kolmogorov-Smirnov Test** | | |
| --- | --- | --- |
|  | | (CEJ-AV) distance |
| N | | 60 |
| Normal Parameters^a,b^ | Mean | 698.4141 |
|  | Std. Deviation | 216.96628 |
| Most Extreme Differences | Absolute | .110 |
|  | Positive | .110 |
|  | Negative | -.072 |
| Kolmogorov-Smirnov Z | | .856 |
| Asymp. Sig. (2-tailed) | | .457 |
| a. Test distribution is Normal. | | |
| b. Calculated from data. | | |

**Data Variance Test**

| **Test of Homogeneity of Variances** | | | |
| --- | --- | --- | --- |
| (CEJ-AV) distance | | | |
| Levene Statistic | df1 | df2 | Sig. |
| 1.755 | 19 | 40 | .067 |

***One-way ANOVA***

| **ANOVA** | | | | | |
| --- | --- | --- | --- | --- | --- |
| (CEJ-AV) distance | | | | | |
|  | Sum of Squares | df | Mean Square | F | Sig. |
| Between Groups | 1901774.377 | 19 | 100093.388 | 4.572 | .000 |
| Within Groups | 875613.155 | 40 | 21890.329 |  |  |
| Total | 2777387.532 | 59 |  |  |  |

Post Hoc Test of Tukey

| **(CEJ-AV) distance** | | | | | |
| --- | --- | --- | --- | --- | --- |
| Tukey HSD^a^ | | | | | |
| Group | N | Subset for alpha = 0.05 | | | |
|  |  | 1 | 2 | 3 | 4 |
| S7 | 3 | 461.5643 |  |  |  |
| S3 | 3 | 497.8843 |  |  |  |
| S5 | 3 | 508.9110 | 508.9110 |  |  |
| S1 | 3 | 513.0127 | 513.0127 |  |  |
| PLD7 | 3 | 538.2657 | 538.2657 | 538.2657 |  |
| PL1 | 3 | 569.2470 | 569.2470 | 569.2470 |  |
| PD3 | 3 | 582.2563 | 582.2563 | 582.2563 |  |
| PLD5 | 3 | 603.1397 | 603.1397 | 603.1397 |  |
| PL7 | 3 | 644.9737 | 644.9737 | 644.9737 |  |
| PL5 | 3 | 662.6487 | 662.6487 | 662.6487 |  |
| PD5 | 3 | 680.1370 | 680.1370 | 680.1370 | 680.1370 |
| PLD3 | 3 | 695.9553 | 695.9553 | 695.9553 | 695.9553 |
| PLD1 | 3 | 695.9597 | 695.9597 | 695.9597 | 695.9597 |
| PD1 | 3 | 759.3790 | 759.3790 | 759.3790 | 759.3790 |
| PL3 | 3 | 780.7783 | 780.7783 | 780.7783 | 780.7783 |
| PD7 | 3 | 797.2467 | 797.2467 | 797.2467 | 797.2467 |
| P1 | 3 | 893.6093 | 893.6093 | 893.6093 | 893.6093 |
| P5 | 3 |  | 964.5417 | 964.5417 | 964.5417 |
| P7 | 3 |  |  | 995.5433 | 995.5433 |
| P3 | 3 |  |  |  | 1123.2290 |
| Sig. |  | .084 | .052 | .050 | .067 |
| Means for groups in homogeneous subsets are displayed. | | | | | |
| a. Uses Harmonic Mean Sample Size = 3.000. | | | | | |
