# Supplementary material for: Combination effect of laser diode for photodynamic therapy with doxycycline on a wistar rat model of periodontitis
Source: BMC Oral Health. 2021 Feb 19;21:80. doi: 10.1186/s12903-021-01435-0 (PMC7893773; doi:10.1186/s12903-021-01435-0)
Supplement: Supplementary file 4 — Additional file 4. Meta data-4 Raw Data of Histology Score. [file 12903_2021_1435_MOESM4_ESM.docx]

**Observation of anatomic histopathological preparations**

**1. Calculation of macrophages**

| Group | A (μm) | B (μm) | C (μm) | Mean | STD |
| --- | --- | --- | --- | --- | --- |
| S1 | 4 | 5 | 6 | 5.00 | 1.00 |
| S3 | 3 | 4 | 5 | 4.00 | 1.00 |
| S5 | 3 | 6 | 6 | 5.00 | 1.73 |
| S7 | 4 | 5 | 6 | 5.00 | 1.00 |
| P1 | 10 | 11 | 7 | 9.33 | 2.08 |
| P3 | 7 | 8 | 8 | 7.67 | 0.58 |
| P5 | 8 | 12 | 10 | 10.00 | 2.00 |
| P7 | 7 | 8 | 11 | 8.67 | 2.08 |
| PL1 | 8 | 6 | 5 | 6.33 | 1.53 |
| PL3 | 6 | 6 | 6 | 6.00 | 0.00 |
| PL5 | 6 | 9 | 6 | 7.00 | 1.73 |
| PL7 | 4 | 5 | 8 | 5.67 | 2.08 |
| PD1 | 6 | 5 | 5 | 5.33 | 0.58 |
| PD3 | 4 | 4 | 6 | 4.67 | 1.15 |
| PD5 | 4 | 7 | 4 | 5.00 | 1.73 |
| PD7 | 3 | 3 | 8 | 4.67 | 2.89 |
| PLD1 | 4 | 4 | 4 | 4.00 | 0.00 |
| PLD3 | 3 | 3 | 3 | 3.00 | 0.00 |
| PLD5 | 3 | 5 | 3 | 3.67 | 1.15 |
| PLD7 | 2 | 3 | 4 | 3.00 | 1.00 |

**2. Calculation of lymphocytes**

| Group | A (μm) | B (μm) | C (μm) | Mean | STD |
| --- | --- | --- | --- | --- | --- |
| S1 | 3 | 4 | 4 | 3.67 | 0.58 |
| S3 | 3 | 3 | 3 | 3.00 | 0.00 |
| S5 | 5 | 4 | 5 | 4.67 | 0.58 |
| S7 | 4 | 4 | 4 | 4.00 | 0.00 |
| P1 | 6 | 4 | 5 | 5.00 | 1.00 |
| P3 | 5 | 5 | 6 | 5.33 | 0.58 |
| P5 | 5 | 6 | 5 | 5.33 | 0.58 |
| P7 | 4 | 5 | 4 | 4.33 | 0.58 |
| PL1 | 4 | 6 | 6 | 5.33 | 1.15 |
| PL3 | 8 | 8 | 9 | 8.33 | 0.58 |
| PL5 | 7 | 7 | 8 | 7.33 | 0.58 |
| PL7 | 6 | 6 | 8 | 6.67 | 1.15 |
| PD1 | 5 | 7 | 10 | 7.33 | 2.52 |
| PD3 | 11 | 7 | 11 | 9.67 | 2.31 |
| PD5 | 8 | 8 | 9 | 8.33 | 0.58 |
| PD7 | 7 | 9 | 10 | 8.67 | 1.53 |
| PLD1 | 8 | 8 | 11 | 9.00 | 1.73 |
| PLD3 | 13 | 8 | 14 | 11.67 | 3.21 |
| PLD5 | 10 | 10 | 11 | 10.33 | 0.58 |
| PLD7 | 9 | 9 | 12 | 10.00 | 1.73 |

**3. Calculation of fibroblas**

| Group | A (μm) | B (μm) | C (μm) | Mean | STD |
| --- | --- | --- | --- | --- | --- |
| S1 | 30 | 38 | 30 | 32.67 | 4.62 |
| S3 | 28 | 32 | 38 | 32.67 | 5.03 |
| S5 | 33 | 34 | 36 | 34.33 | 1.53 |
| S7 | 31 | 35 | 37 | 34.33 | 3.06 |
| P1 | 19 | 19 | 18 | 18.67 | 0.58 |
| P3 | 21 | 17 | 17 | 18.33 | 2.31 |
| P5 | 19 | 18 | 21 | 19.33 | 1.53 |
| P7 | 18 | 24 | 23 | 21.67 | 3.21 |
| PL1 | 24 | 26 | 26 | 25.33 | 1.15 |
| PL3 | 26 | 26 | 25 | 25.67 | 0.58 |
| PL5 | 27 | 28 | 29 | 28.00 | 1.00 |
| PL7 | 25 | 30 | 29 | 28.00 | 2.65 |
| PD1 | 21 | 21 | 24 | 22.00 | 1.73 |
| PD3 | 20 | 20 | 23 | 21.00 | 1.73 |
| PD5 | 24 | 25 | 24 | 24.33 | 0.58 |
| PD7 | 19 | 28 | 36 | 27.67 | 8.50 |
| PLD1 | 30 | 26 | 34 | 30.00 | 4.00 |
| PLD3 | 31 | 30 | 31 | 30.67 | 0.58 |
| PLD5 | 33 | 31 | 38 | 34.00 | 3.61 |
| PLD7 | 29 | 33 | 35 | 32.33 | 3.06 |

**Calculation of CEJ-AV**

| Group | A (μm) | B (μm) | C (μm) | Mean | STD |
| --- | --- | --- | --- | --- | --- |
| S1 | 449,972 | 547,739 | 541,327 | 513,013 | 54,689 |
| S3 | 454,714 | 483,221 | 555,718 | 497,884 | 52,074 |
| S5 | 485,886 | 556,313 | 484,534 | 508,911 | 41,057 |
| S7 | 546,323 | 464,484 | 493,886 | 501,564 | 41,456 |
| P1 | 823,261 | 921,321 | 936,260 | 893,614 | 61,384 |
| P3 | 999,939 | 1,131,948 | 1,113,927 | 1,081,938 | 71,583 |
| P5 | 1,011,721 | 899,096 | 982,789 | 964,535 | 58,489 |
| P7 | 956,101 | 1,021,241 | 1,009,279 | 995,540 | 34,675 |
| PL1 | 622,064 | 507,278 | 557,409 | 562,250 | 57,546 |
| PL3 | 813,489 | 812,551 | 716,292 | 780,777 | 55,848 |
| PL5 | 658,931 | 611,051 | 717,964 | 662,649 | 53,553 |
| PL7 | 703,851 | 603,011 | 628,052 | 644,971 | 52,506 |
| PDI | 682,584 | 783,028 | 794,519 | 753,377 | 61,577 |
| PD3 | 633,489 | 553,551 | 561,292 | 582,777 | 44,088 |
| PD5 | 620,008 | 700,351 | 720,064 | 680,141 | 53,001 |
| PD7 | 828,623 | 734,437 | 828,702 | 797,254 | 54,401 |
| PLD1 | 634,746 | 734,155 | 718,991 | 695,964 | 53,556 |
| PLD3 | 729,942 | 635,292 | 722,625 | 695,953 | 52,661 |
| PLD5 | 544,363 | 623,760 | 641,296 | 603,140 | 51,652 |
| PLD7 | 594,889 | 516,920 | 502,988 | 538,266 | 49,530 |

**Note: A: wistar rat-1**

**B: wistar rat-2**

**C: wistar rat-3**
