# Supplementary figures and images for: Combination effect of laser diode for photodynamic therapy with doxycycline on a wistar rat model of periodontitis
Source: BMC Oral Health. 2021 Feb 19;21:80. doi: 10.1186/s12903-021-01435-0 (PMC7893773; doi:10.1186/s12903-021-01435-0)

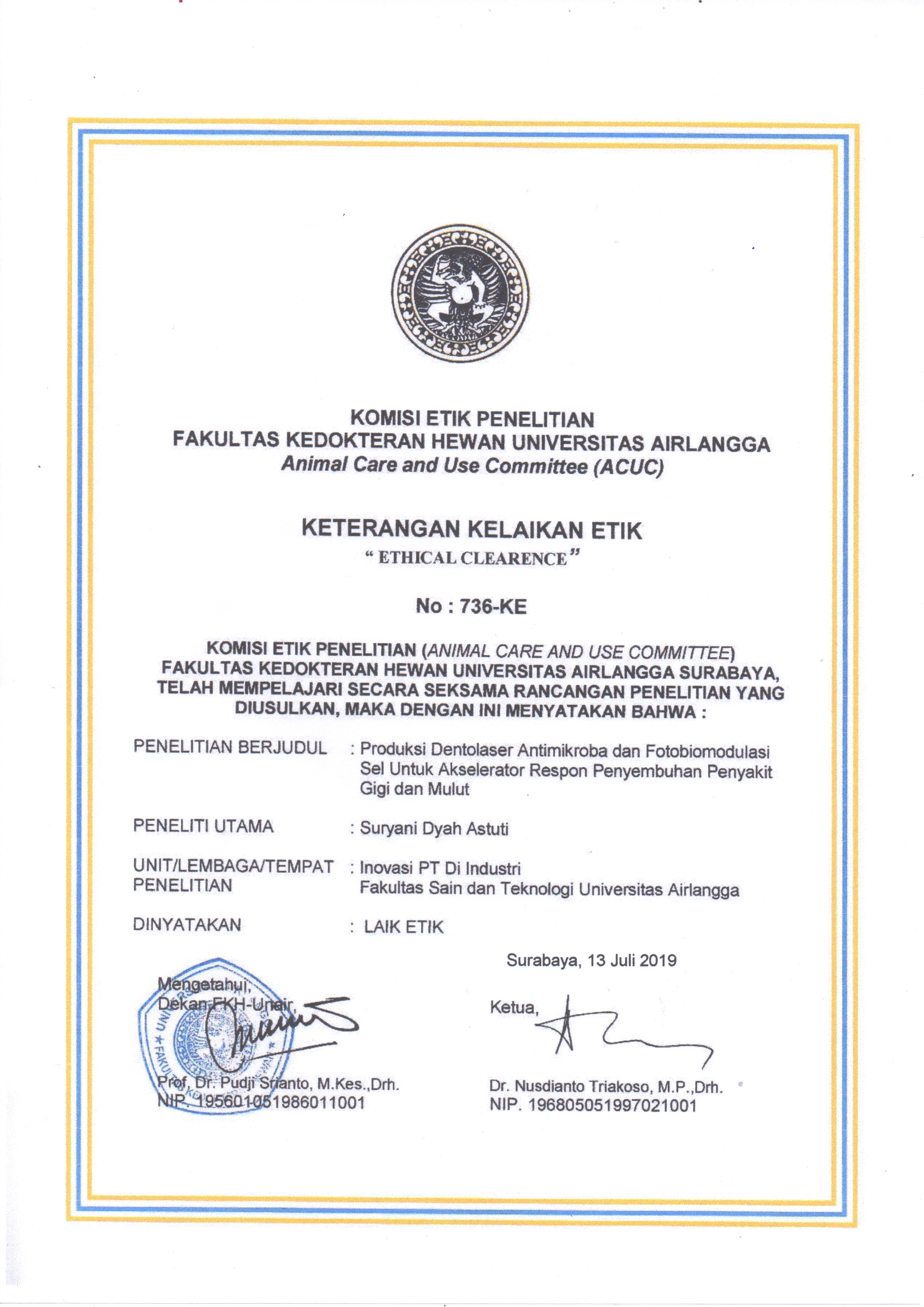

Supplement: Supplementary file 5 — Additional file 5. Ethical clearance. [file 12903_2021_1435_MOESM5_ESM.jpeg]
